# Biosynthesis of Xylariolide D in *Penicillium crustosum* Implies a Chain Branching Reaction Catalyzed by a Highly Reducing Polyketide Synthase

**DOI:** 10.3390/jof8050493

**Published:** 2022-05-09

**Authors:** Sina A. Stierle, Shu-Ming Li

**Affiliations:** Institut für Pharmazeutische Biologie und Biotechnologie, Fachbereich Pharmazie, Philipps-Universität Marburg, 35037 Marburg, Germany; sina.stierle@pharmazie.uni-marburg.de

**Keywords:** *Penicillium*, gene cluster, PKS, heterologous expression, xylariolide D

## Abstract

Fungi are important sources for the discovery of natural products. During the last decades, technological progress and the increasing number of sequenced genomes facilitated the exploration of new secondary metabolites. Among those, polyketides represent a structurally diverse group with manifold biological activities. In this study, we successfully used genome mining and genetic manipulation for functional proof of a polyketide biosynthetic gene cluster from the filamentous fungus *Penicillium crustosum*. Gene activation in the native host and heterologous expression in *Aspergillus nidulans* led to the identification of the *xil* cluster, being responsible for the formation of the 6-methyl-2-pyrone derivative xylariolide D. Feeding with ^13^C-labeled precursors supported the hypothesis of chain branching during the backbone formation catalyzed by a highly reducing fungal polyketide synthase. A cytochrome P450-catalyzed hydroxylation converts the PKS product to the final metabolite. This proved that just two enzymes are required for the biosynthesis of xylariolide D.

## 1. Introduction

Filamentous fungi, including *Penicillium* and *Aspergillus* species, are important sources for natural product (NP) discovery and drug development [1,2]. Polyketide NPs are structurally diverse secondary metabolites. Their backbones are assembled by polyketide synthases (PKSs) and often modified by different tailoring enzymes [3]. Type I fungal PKSs are iterative multifunctional enzymes that mainly use simple acyl building blocks to form a linear carbon chain [4]. In the first step, the PKS is primed with a starter unit, like acetyl-CoA, which is then condensed with an extender unit, e.g., malonyl-CoA. The C-C bond formation is catalyzed by the keto synthase (KS) domain via a decarboxylative Claisen condensation. To allow further chain elongation, the growing polyketide chain bound to the acyl carrier protein (ACP) is relocated to the KS domain. The acyltransferase (AT) domain is responsible for loading a new extender unit onto the ACP domain. Highly reducing (HR) PKSs consist of all reductive domains, i.e., dehydratase (DH), enoyl reductase (ER), and keto reductase (KR), which allow the optional full reduction of the β-keto group after Claisen condensation [4,5].

α-Carbon branches can be introduced into the backbone by methyltransferase (MT) domains using S-adenosyl methionine as the methyl donor or by extender units with α-substituents like methylmalonyl-CoA [6]. Several enzymes that catalyze ß-branching by condensation at the ß-position to the carbonyl group of the ACP-bound polyketide chain have been reported, including RhiE with a B domain for the branching reaction [7,8].

*Penicillium crustosum* (*P. crustosum*) PRB-2 is a marine fungus isolated from deep-sea sediment [9]. Mining the genome of PRB-2 and gene prediction with antiSMASH [10] indicated the presence of at least 58 putative biosynthetic gene clusters for secondary metabolites. Previous work with *P. crustosum* PRB-2 revealed that only a few of these gene clusters, such as those of peniphenones, penilactones, and their precursors, are expressed under laboratory conditions [11,12]. For uncovering NPs encoded by these silent or cryptic genes, gene activation in the producer and heterologous expression in a well-studied host have proven reliable genetic approaches [13,14], which were also successfully used for gene identification in PRB-2 [15]. To enhance genetic manipulation, a *pyrG* deficient strain FK15 was created in a previous study [16]. This strain was further modified by deletion of *pcr4870*, coding for the DNA repair protein subunit Ku70, to improve gene targeting efficiency. The resulting *P. crustosum Δpcr4870ΔpyrG* strain JZ02 can be conveniently used for gene deletion and activation experiments [17].

In this study, combined genetic approaches, including activation of genes in the native host as well as heterologous expression in *Aspergillus nidulans* (*A. nidulans*), were used to elucidate the function of a silent gene cluster from *P. crustosum* (Table S1). This led to the isolation of xylariolide D (**1**) and its congener prexylariolide D (**2**). Incorporation of ^13^C-labeled precursors proved that the methyl residue of xylariolide D is derived from acetate, indicating that the highly reducing PKS also catalyzed a branching reaction during the biosynthesis.

## 2. Materials and Methods

### 2.1. Plasmids and Strains

Plasmids and strains used in this study are listed in Tables S2 and S3. All plasmids were constructed by using an assembly approach based on homologous recombination in *Saccharomyces cerevisiae* HOD114-2B [18]. For heterologous expression in *A. nidulans* LO8030 [19], an expression vector was created by using pJN17 as a template for the amplification of *Amp*/*URA3*, *wA* flanking regions, and *gpdA(p)* [20]. Instead of *AfriboB*, *AfpyrG* was amplified from the plasmid pYH-wA-pyrG [21] and used as a selection marker for all strains. The expression vector was sequenced to confirm correct assembly and linearized with *Sfo*I. The genes for expression were amplified from genomic DNA (see Supplementary Materials for detail).

Plasmids for the activation of genes in the native host *P. crustosum* were assembled likewise: *Amp*/*URA3*, flanking regions, *AfpyrG*, *gpdA(p),* and genes of interest were amplified by PCR and assembled via yeast homologous recombination. *E. coli* was used to amplify plasmids after construction in *S. cerevisiae* (see Supplementary Materials for more detail). Genetic manipulation in *E. coli* was carried out as described before [22].

### 2.2. Transformation of Fungal Strains and Confirmation of Integration

*A. nidulans* LO8030 and *P. crustosum* JZ02 were used for PEG-mediated protoplast transformation according to protocols described previously [12]. For selection of recombinant strains, media lacking uridine and uracil were used. Correct integration of the constructs into the genome was verified by PCR amplification with genomic DNA as template (see Supplementary Materials for more details). Afterward, transformants were cultivated, and the extracts were submitted to LC-MS analysis.

### 2.3. Cultivation of Fungal Strains, Extraction and Isolation of Secondary Metabolites

For LC-MS analysis, 50 mL of liquid glucose minimal medium [3] (GMM, see Supplementary Materials for more details) and Czapek-Dox (CD) medium were inoculated with spores and cultivated at 25 °C in the dark under static conditions. After 14 days, 1 mL of liquid culture was extracted with the same volume of EtOAc twice. After evaporation of the solvent, the residue was dissolved in 95% MeOH and submitted to LC-MS analysis (see Supplementary Materials for details).

Large-scale cultivation for the isolation of metabolites was carried out in 3 L GMM or Czapek-Dox (CD) medium. Compounds **1** and **2** were isolated from culture broths of *P. crustosum* SSt12, and compounds **2** and **3** were isolated from *A. nidulans* SSt04 and *A. nidulans* SSt26. After extraction with the same volume of EtOAc twice, the compounds were isolated by silica gel chromatography and semi-preparative HPLC (see Supplementary Materials for details).

### 2.4. Feeding with Prexylariolide D (***2***)

Cultivation was performed using 10 mL liquid GMM in 50 mL flasks at 25 °C in the dark under static conditions. Prexylariolide D (**2**) was dissolved in MeOH and added in a final concentration of 0.5 mM to 3-day-old cultures of *A. nidulans* SSt28 and *A. nidulans* LO8030. For LC-MS analysis, 0.5 mL samples were taken after 10 days of cultivation and extracted with the same volume of EtOAc. 

### 2.5. Incorporation of ^13^C-Labeled Precursors

[2-^13^C] and [1,2-^13^C] acetate were purchased from Cambridge Isotope Laboratories, Inc. (Tewksbury, MA, USA). To isolate ^13^C enriched **1** and **2** from *P. crustosum* SSt12, 2 × 250 mL of liquid GMM were inoculated with spores and kept at 25 °C for 3 days. For each flask, 75 mg of [2-^13^C] and [1,2-^13^C] acetate were dissolved in 1 mL of GMM and added to the cultures. Isolation was performed after 10 days of cultivation on a semi-preparative HPLC (see Supplementary Materials for more detail), leading to approximately 3 mg of analytically pure **1** and **2**.

## 3. Results

### 3.1. Bioinformatic Analysis

**Figure 1:**
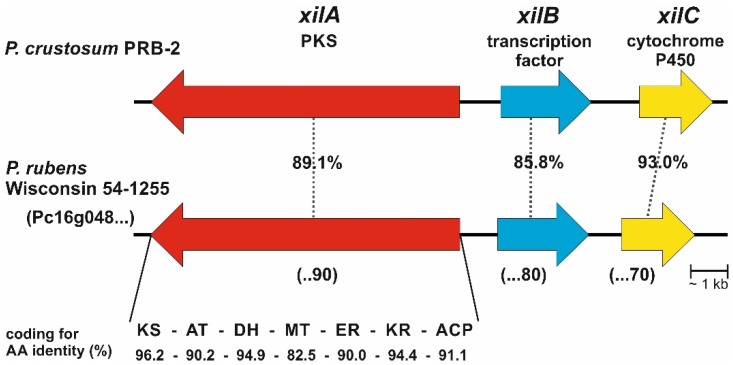
Comparison of *xil* cluster from *P. crustosum* PRB-2 with its ortholog from *P. rubens* Wisconsin 54-1255. Accession numbers for genes from *P. rubens* are given in parentheses.

One of the putative silent clusters from *P. crustosum* PRB-2, termed the *xil* cluster in this study (Figure 1), attracted our attention. The cluster of interest comprises three genes coding for an HR-PKS XilA, a fungal-specific transcription factor XilB, and a cytochrome P450 enzyme XilC. BLAST search revealed that *xilA* and *xilC* share identical nucleotide sequences with those coding for KAF7517450.1 and KAF7517449.1 from *Penicillium crustosum* G10, respectively. An ortholog for *xilB* was not reported for strain G10. BLAST search also indicated the presence of a similar gene cluster in *Penicillium rubens* Wisconsin 54-1255 (=DSM 1075). XilA, XilB, and XilC show high sequence identities of 89.1, 85.8, and 93.0% to Pc16g04890, Pc16g04880, and Pc16g04870 from *P. rubens,* respectively (Figure 1) [23]. The polyketide synthases XilA and Pc16g04890 share the same domain structure comprising KS-AT-DH-MT-ER-KR-ACP with sequence identities from 82% to 96% on the amino acid level between the respective domains. In addition, XilA shows a sequence identity of 52.2% to the HR-PKS Sol1 (D7UQ44.1) from *Alternaria solani* with the same domain structure. This PKS assembles a 3-methyl-2-pyrone derivative as the PK scaffold [24,25,26].

### 3.2. Activation of Transcription Factor xilB and PKS xilA in P. crustosum

**Figure 2:**
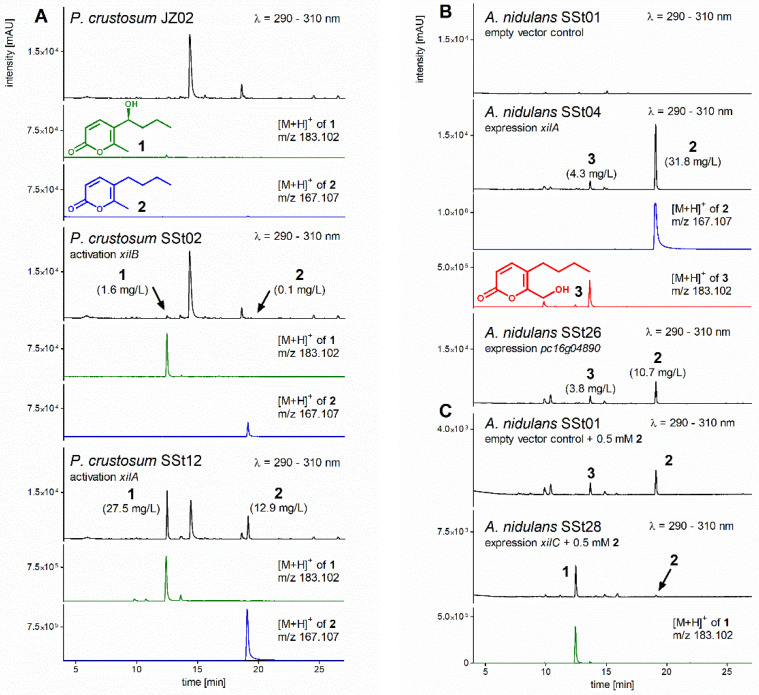
LC-MS analysis of fungal extracts. (**A**) Activation of the transcription factor *xilB* and polyketide synthase *xilA* in *P. crustosum*, (**B**) expression of *xilA* and its ortholog *pc16g04890* in *A. nidulans*, (**C**) adding **2** to *A. nidulans* with and without *xilC* expression. Compounds **1**–**3** with their EICs are highlighted in green, blue, and red, respectively. A tolerance range of ±0.005 was used for ion detection. Product yields after gene activation or expression are given in parentheses under the compound numbers.

To identify the product of the *xil* cluster, we activated the transcription factor *xilB* by cloning the *gpdA(p)* promoter from *A. nidulans* [16] in front of its coding region. The resulting linearized construct pSSt02 was introduced into *P. crustosum* JZ02 via PEG-mediated protoplast transformation, as mentioned in Section 2.2. The obtained transformant *P. crustosum* SSt02 (*AfpyrG*::*gpdA(p)*::*xilB*) was verified via PCR (Figure S6) and cultivated in GMM for 14 days. In comparison to the control strain JZ02, LC-MS analysis revealed the presence of a new compound **1** with a [M+H]^+^ ion at *m/z* 183.1019 in the extract of SSt02 (Figure 2A). However, the low quantity prohibited us from structure elucidation. Therefore, we used the same strategy as for *xilB* to activate the PKS gene *xilA*, resulting in strain *P. crustosum* SSt12 (*AfpyrG*::*gpdA(p)*::*xilA*). LC-MS analysis indicated an at least 17-fold higher accumulation of **1** in the *xilA* overexpression strain SSt12 than in the strain SSt02 after *xilB* activation (Figure 2A). Furthermore, another new compound **2** was detected with a [M+H]^+^ ion at *m/z* 167.1067. Product yields of 27.5 and 12.9 mg/L were calculated for **1** and **2** in SSt12, respectively.

### 3.3. Heterologous Expression of xilA and pc16g04890

To identify the XilA product by heterologous expression in *A. nidulans*, *xilA* was cloned into the expression vector under the control of the constitutive *gpdA(p)* promoter and expressed in LO8030, resulting in the transformant *A. nidulans* SSt04. Compared to the control strain *A. nidulans* SSt01, carrying the empty expression vector, SSt04 produced **2** and a new compound **3** with [M+H]^+^ ions at *m/z* 167.1071 and 183.1016, respectively (Figure 2B). Compounds **1** and **3** share the same [M+H]^+^ ion but differ from each other in retention times. With a larger molecular mass of 16 daltons, they are likely hydroxylated products of **2**.

To confirm that the PKS Pc16g04890 from *P. rubens* Wisconsin 54-1255 produces the same compounds as XilA, its genomic sequence was also expressed in *A. nidulans*. Cultivation of the obtained transformant SSt26 led indeed to the accumulation of **2** and **3** (Figure 2B and Figure S4).

### 3.4. Structure Elucidation

**Figure 3:**
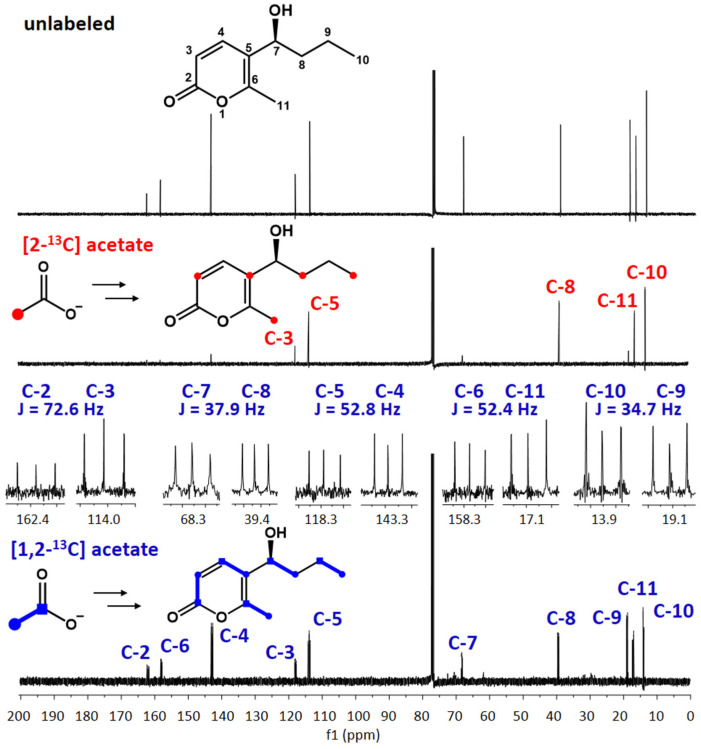
^13^C-NMR spectra of xylariolide D (**1**) without and with ^13^C-labeled acetate.

**Figure 4:**
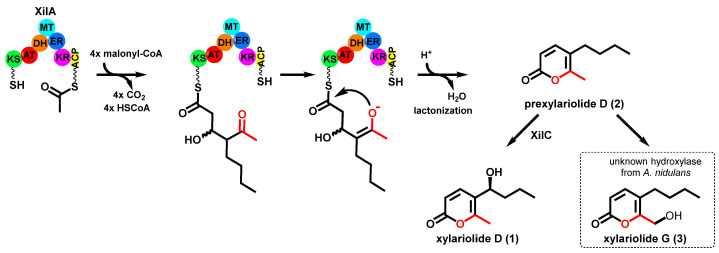
Proposed biosynthetic pathway for the formation of xylariolide D in *P. crustosum,* including a branching step during the polyketide chain elongation as well as formation of xylariolide G in *A. nidulans*.

The obtained analytically pure compounds **1**–**3** were subjected to NMR analysis, including ^1^H-NMR, ^13^C-NMR, HMBC, and HSQC (Figures S7–S20). Interpretation of the NMR spectra and comparison with data in the literature proved **1** and **2** to be xylariolide D [27] and its non-hydroxylated precursor 5-butyl-6-methyl-2H-pyran-2-one (named prexylariolide D in this study) [28], respectively (see Figure 2 and Figure 3 for structures). ECD spectrum (Figure S23) confirmed the S-configuration of **1**, as reported previously [27]. It can be speculated that **2** is the PKS (XilA) product and hydroxylated to **1** by the cytochrome P450 enzyme XilC in *P. crustosum* (Figure 4). Comparison of the NMR data of **2** and **3** revealed that the signals at *δ*_H_ 2.22 (s, 3H) and *δ*_C_ 17.2 ppm for the methyl group at the 2-pyrone ring of **2** disappeared in the spectra of **3**. Instead, signals for a methylene group were observed at *δ*_H_ 4.45 (s, 2H) and *δ*_C_ 59.0 ppm (Figures S12, S13, S16, and S17), indicating the conversion of the methyl to a hydroxymethyl group in **3**. This was also confirmed by HSQC and HMBC analyses. Since **3** (named xylariolide G) is not produced by the native host *P. crustosum*, even after activation of *xilA* or *xilB*, we assume that the conversion of **2** to **3** in *A. nidulans* SSt04 is catalyzed by an enzyme from the host strain, as observed for a methylated isocoumarine after heterologous expression of a PKS gene [22].

### 3.5. Heterologous Expression of xilC and Feeding of Prexylariolide D

To provide evidence for the conversion of **2** to **1** in *P. crustosum* by the cytochrome P450 XilC and **2** to **3** in *A. nidulans* by a host enzyme, we created a *xilC* overexpression strain *A. nidulans* SSt28. Compound **2** (0.5 mM) was added to the three-day-old cultures of SSt28 and the control strain LO8030. As shown in Figure 2C, **2** was almost completely converted to **1** in SSt28 and about 30% to **3** in the control strain. These results proved that **2** was hydroxylated by XilC at the side chain and by an unknown enzyme from *A. nidulans* at the methyl group of the 2-pyrone ring (Figure 4).

### 3.6. Feeding with ^13^C-Labeled Precursors

To clarify whether the methyl residue at the 2-pyrone ring of **1** is derived from L-methionine or acetate, feeding experiments with [2-^13^C] and [1,2-^13^C] acetate were conducted in *P. crustosum* SSt12. Both **1** and **2** were isolated and subjected to ^13^C NMR analysis. As shown in Figure 3 and Figure S21, incorporation of ^13^C with enrichments between 5.0 and 9.0% (Table S5) was observed for C3, C5, C8, C10, and C11 in the spectra of both **1** and **2** isolated after [2-^13^C] acetate feeding. These results proved unequivocally the incorporation of five acetate units in their structures and acetate as the origin of both methyl groups. This conclusion was also supported by the observed ^13^C-^13^C coupling pattern in the ^13^C NMR spectra of **1** and **2** (Figure 3 and Figure S22, Table S5) after feeding with [1,2-^13^C] acetate.

## 4. Discussion

Xylariolide D was isolated from different fungal strains, e.g., *Xylaria* sp. NCY2, *Neodidymelliopsis* sp., and *Dictyosporium digitatum* [29,30,31]. However, the gene cluster responsible for its biosynthesis has not been described previously. Based on the results from feeding and incorporation experiments, we postulate a mechanism for the formation of prexylariolide D (**2**) catalyzed by the type I iterative HR-PKS XilA (Figure 4). During the biosynthesis, the linear polyketide chain is branched by the addition of an acetyl unit. Product release is achieved via 2-pyrone ring formation accompanied by water elimination. As shown in Figure 1, a putative MT domain was predicted for XilA and Pc16g04890. However, activation of *xilA* in *P. crustosum* and heterologous expression of both genes in *A. nidulans,* as well as feeding experiments, proved the accumulation of metabolites without the involvement of a methyl transfer reaction. It seems that the MT domain in both PKSs is inactive or has another function.

As mentioned in Section 3.1, bioinformatic analysis indicates that *xilB* encodes a fungal-specific transcription factor. By *xilB* activation, we were able to slightly increase xylariolide D (**1**) production in *P. crustosum*. However, activation of the PKS gene *xilA* not only resulted in the formation of **2**, but also in a significantly higher yield of product **1** compared to that after *xilB* activation. Obviously, XilB had less influence on the regulation of xylariolide D biosynthesis. It is possible that the entire *xil* cluster is under the control of other systems like the global regulation of secondary metabolism. Furthermore, the production of both compounds **1** and **2** in *P. crustosum* SSt12 after overexpression of *xilA* indicates that the formation of the polyketide scaffold, and not the hydroxylation, is the limiting step in the biosynthesis of xylariolide D.

As shown in Figure 2, feeding of prexylariolide D (**2**) led to the accumulation of xylariolide D (**1**) in the *xilC* overexpression strain *A. nidulans* SSt28 and xylariolide G (**3**) in the control *A. nidulans* SSt01. Dihydroxylated derivative was not detected in SSt28, proving the higher substrate specificity of XilC toward its natural substrate **2** than the unknown hydroxylase from *A. nidulans*. Only in the absence of XilC, prexylariolide D (**2**) was converted to **3** by the hydroxylase from *A. nidulans*.

## 5. Conclusions

In conclusion, we elucidated the function of a silent gene cluster for the biosynthesis of xylariolide D (**1**) in two fungal strains by gene activation in the native strain and heterologous expression in a well-studied host. Activation of the PKS gene *xilA* led to the accumulation of **1** with a much higher yield than the activation of the putative transcription factor *xilB*. Feeding experiments confirmed that XilC catalyzes the hydroxylation of prexylariolide D (**2**) to **1**. The incorporation of ^13^C-labeled precursors provides clear evidence for acetate as the origin of the methyl group at the 2-pyrone ring. Further investigation, such as the biochemical characterization or elucidation of the XilA structure, will gain deeper insight into the unusual formation of the 5-alkyl-6-methyl-2-pyrone backbone.

## Data Availability

Not applicable.

## Supplementary Materials

The following supporting information can be downloaded at: https://www.mdpi.com/article/10.3390/jof8050493/s1. Table S1. xil cluster in P. crustosum and P. rubens. Table S2. Strains used in this study. Table S3. Oliogonucleotide primers and plasmids used in this study. Table S4. NMR data of compounds 1, 2 and 3 in CDCl3. Table S5. Enrichments and coupling constants in 1 and 2 after feeding with 13C labeled precursors. Figure S1. Schematic representation of plasmid creation. Figure S2. Schematic representation of gene integration into the wA PKS locus of A. nidulans LO8030. Figure S3. Verification of plasmid pSSt04 and correct integration in A. nidulans SSt04 transformants. Figure S4. Verification of plasmid pSSt26 and correct integration in A. nidulans SSt26 transformant. Figure S5. Verification of plasmid pSSt28 and correct integration in A. nidulans SSt28 transformants. Figure S6. Verification of plasmids and correct gene integration in P. crustosum transformants. Figure S7. 1H-NMR of compound 1 in CDCl3 (500 MHz). Figure S8. 13C-NMR of compound 1 in CDCl3 (125 MHz). Figure S9. HSQC of compound 1 in CDCl3. Figure S10. HMBC of compound 1 in CDCl3. Figure S11. H,H-COSY of compound 1 in CDCl3. Figure S12. 1H-NMR of compound 2 in CDCl3 (500 MHz). Figure S13. 13C-NMR of compound 2 in CDCl3 (125 MHz). Figure S14. HSQC of compound 2 in CDCl3. Figure S15. HMBC of compound 2 in CDCl3. Figure S16. 1H-NMR of compound 3 in CDCl3 (500 MHz). Figure S17. 13C-NMR of compound 3 in CDCl3 (125 MHz). Figure S18. HSQC of compound 3 in CDCl3. Figure S19. HMBC of compound 3 in CDCl3. Figure S20. H,H-COSY of compound 3 in CDCl3. Figure S21. 13C-NMR of compound 2 in CDCl3, enriched with [2-13C] acetate (125 MHz). Figure S22. 13C-NMR of compound 2 in CDCl3, enriched with [1,2-13C] acetate (125 MHz). Figure S23. ECD-spectrum of compound 1 (0.7 mg/mL) in MeOH. Ref. [32] is cited in Supplementary Materials.
