# Supplementary material for: Biosynthesis of Xylariolide D in Penicillium crustosum Implies a Chain Branching Reaction Catalyzed by a Highly Reducing Polyketide Synthase
Source: J Fungi (Basel). 2022 May 9;8(5):493. doi: 10.3390/jof8050493 (PMC9147667; doi:10.3390/jof8050493)
Supplement: Supplementary file 1 [file jof-08-00493-s001.zip › jof-1713420-supplementary.pdf]

SUPPLEMENTARY INFORMATION

Biosynthesis of Xylariolide D in *Penicillium crustosum* Implies a Branching Reaction Catalyzed by a Highly Reducing Polyketide Synthase

Sina A. Stierle and Shu-Ming Li*
Institut für Pharmazeutische Biologie und Biotechnologie, Fachbereich Pharmazie, Philipps-Universität Marburg, Robert-Koch-Straße 4, Marburg 35037, Germany

Table of contents

Experimental procedures **4**
Bioinformatic tools and sequence analysis 4
Strains, growth conditions and media for cultivation 4
Genomic DNA isolation 4
PCR amplification, gene cloning and plasmid construction 4
LC-MS analysis 5
Large scale fermentation, metabolite extraction, and isolation 5
NMR analyses 5
Circular dichroism (CD) spectroscopic analysis of compound **1** 5
Physiochemical properties of the compounds described in this study 5
Supplementary Tables **6**
Table S1. *xil* cluster in *P. crustosum* and *P. rubens*. 6
Table S2. Strains used in this study. 7
Table S3. Oliogonucleotide primers and plasmids used in this study. 8
Table S4. NMR data of compounds **1**, **2** and **3** in $CDCl_3$. 10
Table S5. Enrichments and coupling constants in **1** and **2** after feeding with ^{13}C labeled precursors. 11
Supplementary Figures **12**
Figure S1. Schematic representation of plasmid creation. 12
Figure S2. Schematic representation of gene integration into the *wA* PKS locus of *A. nidulans* LO8030. 13
Figure S3. Verification of plasmid pSSt04 and correct integration in *A. nidulans* SSt04 transformants. 14
Figure S4. Verification of plasmid pSSt26 and correct integration in *A. nidulans* SSt26 transformant. 15
Figure S5. Verification of plasmid pSSt28 and correct integration in *A. nidulans* SSt28 transformants. 16
Figure S6. Verification of plasmids and correct gene integration in *P. crustosum* transformants. 17
Figure S7. ^{1}H-NMR of compound **1** in $CDCl_3$ (500 MHz). 18
Figure S8. ^{13}C-NMR of compound **1** in $CDCl_3$ (125 MHz). 19
Figure S9. HSQC of compound **1** in $CDCl_3$. 20
Figure S10. HMBC of compound **1** in $CDCl_3$. 21
Figure S11. H,H-COSY of compound **1** in $CDCl_3$. 22
Figure S12. ^{1}H-NMR of compound **2** in $CDCl_3$ (500 MHz). 23
Figure S13. ^{13}C-NMR of compound **2** in $CDCl_3$ (125 MHz). 24
Figure S14. HSQC of compound **2** in $CDCl_3$. 25

Figure S15. HMBC of compound **2** in $CDCl_3$. 26
Figure S16. ^{1}H-NMR of compound **3** in $CDCl_3$ (500 MHz). 27
Figure S17. ^{13}C-NMR of compound **3** in $CDCl_3$ (125 MHz). 28
Figure S18. HSQC of compound **3** in $CDCl_3$. 29
Figure S19. HMBC of compound **3** in $CDCl_3$. 30
Figure S20. H,H-COSY of compound **3** in $CDCl_3$. 31
Figure S21. ^{13}C-NMR of compound **2** in $CDCl_3$, enriched with [2-^{13}C] acetate (125 MHz). 32
Figure S22. ^{13}C-NMR of compound **2** in $CDCl_3$, enriched with [1,2-^{13}C] acetate (125 MHz). 33
Figure S23. ECD-spectrum of compound **1** (0.7 mg/mL) in MeOH. 34
References 35

Experimental procedures

Bioinformatic tools and sequence analysis

The genomic DNA sequence for the *xil* cluster from *Penicillium crustosum* PRB-2 (Table S1) is deposited at GenBank under the accession number OM831135. Gene cluster prediction was carried out by using AntiSMASH 6.0 (https://fungismash.secondarymetabolites.org/#!/start) [1]. BLAST searches (https://blast.ncbi.nlm.nih.gov/Blast.cgi) were used for sequence analysis and comparison with known entries. Domain structure prediction for XilA and Pc16g04890 was carried out with BLAST, AntiSMASH 6.0, PKS/NRPS Analysis (http://nrps.igs.umaryland.edu/) and SBSPKSv2 (http://202.54.226.228/~pksdb/sbspks_updated/master.html).
SnapGene software (www.snapgene.com) was used for plasmid illustration.

Strains, growth conditions and media for cultivation

All strains used in this study are summarized in Table S2. Fungal strains were cultivated in liquid glucose minimal medium [2] (GMM, 1.0% glucose, 50 mL/L salt solution, 1 mL/L trace element solution) supplemented with the required selection markers (uridine, uracil, riboflavin or pyridoxine). Salt solution (w/v) contains 12% $NaNO_3$, 1.04% KCl, 1.04% $MgSO_4 \cdot 7H_2O$, 3.04% KH_2PO_4. Trace element solution (w/v) consists of 2.2% $ZnSO_4 \cdot 7H_2O$, 1.1% H_3BO_3, 0.5% $MnCl_2 \cdot 4H_2O$, 0.16% $FeSO_4 \cdot 7H_2O$, 0.16% $CoCl_2 \cdot 5H_2O$, 0.16% $CuSO_4 \cdot 5H_2O$, 0.11% $(NH_4)_6Mo_7O_{24} \cdot 4H_2O$, 5% Na_4EDTA.[1] For GMM plates, 1.5% agar was added prior to autoclaving. For sporulation and transformation, *P. crustosum* and *P. rubens* were grown at 25°C on plates and *A. nidulans* at 37°C. Czapek-Dox (CD) medium was obtained from Becton and Dickinson. Small scale cultivation was performed in 250 ml flasks containing 50 mL of media, which were inoculated with 5 µl of a spore suspension of the strain of interest. Fungal strains were cultivated in the dark at 25°C under static conditions. Samples for LC-MS analysis were taken after 14 days.

Saccharomyces cerevisiae HOD114-2B was used for cloning *via* homologous recombination using uracil as selection marker. It was grown at 30°C in YPD medium (1% yeast extract, 2% peptone, 2% glucose) or on YPD plates containing 1.5% agar. Selection was performed with synthetic complete medium without uracil (SC-Ura, 6.7 g/L yeast nitrogen base with ammonium sulfate, 0.65 g/L CSM-His-Leu-Ura, 0.02 g/L histidine and 0.06 g/L leucine).

Escherichia coli DH5α from Thermo Fisher Scientific Inc. (Waltham, MA, United States) and *Escherichia coli* XL10-gold from Agilent Technologies, Inc. (Santa Clara, CA, United States) were used for plasmid propagation. They were grown on solid or in liquid LB (lysogeny broth) at 37°C. For selection of recombinant *E. coli* strains, 50 µg/mL ampicillin w used.

Genomic DNA isolation

For genomic DNA isolation, *A. nidulans*, *P. crustosum*, and *P. rubens* strains were cultivated in 5 mL of liquid GMM at 25°C for 2-3 days. Mycelia were collected on sterilized filter paper and transferred into 2 mL Eppendorf tubes. Glass beads (2.85 mm in diameter) and 700 µL LETS solution (20 mM EDTA pH 8.0, 0.5% (w/v) SDS, 0.1 M LiCl, and 10 mM Tris-HCl pH 8.0) were added and mycelia were crushed using Minilys Homogenizer (Bertin Technologies, Montigny-le-Bretonneux. France) for 200 s at full speed. After centrifugation, the supernatant was transferred to another tube and mixed with 700 µL phenol:chloroform:isoamyl alcohol, followed by centrifugation at 13,000 rpm for 10 min. The aqueous phase was transferred into a tube containing 700 µL chloroform:isoamyl alcohol, followed by centrifugation at 13,000 rpm for 10 min. DNA was precipitated by addition of 900 µL absolute cold ethanol. After centrifugation at 13,000 rpm and 4°C for 30 min, the supernatant was dismissed and the remaining DNA pellet was washed with 400 µL 70% ethanol and dissolved in 200 µL water.

PCR amplification, gene cloning and plasmid construction

Plasmids and oligonucleotide sequences for PCR amplification used in this study are listed in Table S3. Primers were synthesized by Microsynth Seqlab GmbH (Göttingen, Germany). PCR amplification was achieved using Phusion® High-Fidelity DNA polymerase from New England Biolabs (NEB) on a T100TM Thermal cycler from Bio-Rad. Genes were amplified by PCR as DNA fragments containing an overhang of about 30 bp to each other. All plasmids were constructed using *Saccharomyces cerevisiae* HOD114-2B. An assembly approach based on homologous recombination was used as described previously [3].

LC-MS analysis

For LC-MS analysis, 1 mL of liquid culture was extracted with 1 mL of EtOAc twice. After evaporation, the residue was dissolved in 95% MeOH and measured on an Agilent HPLC 1260 series system with a photo diode array detector and a Bruker micro TOF QIII mass spectrometer by using a VDSpher PUR 100 C18-M-SE column (150 x 2.0 mm, 3 µm, VDS optilab Chromatographietechnik GmbH, Berlin, Germany). A linear gradient from 5 – 100 % acetonitrile in water, containing 0.1 % formic acid, in 30 min at a flow rate of 0.3 mL/min was used for LC-MS analysis. The parameters of the MS device were set as electrospray positive ion mode for ionization, capillary voltage of 4.5 kV and collision energy of 8.0 eV.

Large scale fermentation, metabolite extraction, and isolation

For the isolation of compounds **2** and **3** from *A. nidulans* SSt04, the strain was cultivated in six 2 L Erlenmeyer flasks containing 500 ml of liquid GMM each at 25°C in the dark under static conditions for 14 days. Extraction was performed twice using one volume of EtOAc. Evaporation of the solvent under reduced pressure gave a crude extract of 0.2 g, which was then purified by silica gel column chromatography using petroleum ether/EtOAc as solvents (4:1, 2:1, 1:1, 1:2, 1:4 v/v). After further purification on Agilent HPLC 1260 series system by using a semi-preparative MultoHigh 100-5 SI column (250 x 10 mm, CS-Chromotographie Service GmbH, Langerwehe, Germany, CH_2Cl_2/MeOH =98/2, flow rate: 2.0 mL/min, λ = 300 nm), 6 mg of **2** and 5 mg of **3** were obtained. Isolation of compound **1** from *P. crustosum* SSt12 and **2** from *A. nidulans* SSt26 and *P. crustosum* SSt12 in CD medium was performed accordingly.

NMR analyses

NMR spectra of the purified compounds were recorded on a JEOL ECA-500 spectrometer (JEOL, Akishima, Tokyo, Japan). All samples were dissolved in $CDCl_3$. Spectra were processed using MestReNova 9.0.0 (Mestrelab Research, Santiago de Compostella, Spain). Chemical shifts are referenced to those of the solvents signals and given in Table S4.

Circular dichroism (CD) spectroscopic analysis of compound 1

CD spectrum was taken on a J-815 CD spectrometer (Jasco Deutschland GmbH, Pfungstadt, Germany). The sample was dissolved in MeOH and measured in the range of 200–400 nm by using a 1 mm path length quartz cuvette (Hellma Analytics, Müllheim, Germany). The CD spectrum of compound **1** is given in Figure S12.

Physiochemical properties of the compounds described in this study

Xylariolide D (**1**): colorless volatile oil. HRMS (ESI) m/z: $[M+H]^+$ Calcd for $C_{10}H_{15}O_3$ 183.1016; Found 183.1019. ECD (0.7 mg/mL, MeOH) λ_{max} (Δε) 301 (-6.13), 230 (+7.33), 205 (−64.71) nm

Prexylariolide D (**2**): colorless volatile oil. HRMS (ESI) m/z: $[M+H]^+$ Calcd for $C_{10}H_{15}O_2$ 167.1067; Found 167.1075

Xylariolide G (**3**): colorless volatile oil. HRMS (ESI) m/z: $[M+H]^+$ Calcd for $C_{10}H_{15}O_3$ 183.1016; Found 183.1021

Supplementary Tables

Table S1. *xil* cluster in *P. crustosum* and *P. rubens*.

Penicillium crustosum PRB-2				*Penicillium crustosum* G10	*Penicillium rubens* Wisconsin 54-1255
protein	gene	length [aa]	Putative function	Accession Nr (length/identity)	Accession Nr (length /identity)
XilA	*pcr4475*	2492	PKS	KAF7517450.1 (2492 aa/ 89.1%)	XP_002560839.1 (2464 aa/ 89.1%)
XilB	*pcr4474*	654	Transcription factor	-	XP_002560838.1 (671 aa / 85.8%)
XilC	*pcr4473*	572	Cytochrome P450	KAF7517449.1 (572 aa / 100%)	XP_002560837.1 (572 aa / 93.0%)

Table S2. Strains used in this study.

strain	genotype	created with plasmid	reference
E. coli			
DH5α	*F– endA1 glnV44 thi-1 recA1 relA1 gyrA96 deoR nupG purB20 φ80dlacZΔM15 Δ(lacZYA-argF)U169, hsdR17(rK–mK+), λ⁻*		
XL10 gold	*TetrD(mcrA)183 D(mcrCB-hsdSMR-mrr)173 endA1 supE44 thi-1 recA1 gyrA96 relA1 lac Hte [F´ proAB lacIqZDM15 Tn10 (Tetr) Amy Camr]*		
S. cerevisiae			
HOD114-2B	*MATα ura3-52 his3Δ1 leu2-3112*		[3]
A. nidulans			
LO8030	*pyroA4, riboB2, AfpyrG89, nkuA::argB*, deletion of secondary metabolite clusters: (AN7804-AN7825)Δ, (AN2545-AN2549)Δ, (AN1039-AN1029)Δ, (AN10023-AN10021)Δ, (AN8512-AN8520)Δ, (AN8379-AN8384)Δ, (AN9246-AN9259)Δ, (AN7906-AN7915)Δ, (AN6000-AN6002)Δ.		[4]
SSt01	*wA-PKS*::*gpdA(p)-AfpyrG* in *A. nidulans* LO8030	pSSt05	this study
SSt04	*wA-PKS*::*gpdA(p)-xilA* + 999bp 3'UTR-Af*pyrG* in *A. nidulans* LO8030	pSSt04	this study
SSt26	*wA-PKS::gpdA(p)- Pc16g04890* + 501bp 3'UTR-*AfpyrG* in *A. nidulans* LO8030	pSSt26	this study
SSt28	*wA-PKS::gpdA(p)- xilC* + 501bp 3'UTR-*AfpyrG* in *A. nidulans* LO8030	pSSt28	this study
P. crustosum			
PRB-2	wildtype		[5]
JZ02	*ΔpyrGΔku70*		[6]
SSt02	*AfpyrG::gpdA(p)::xilB* in *P. crustosum* JZ02	pSSt02	this study
SSt12	*AfpyrG::gpdA(p)::xilA* in *P. crustosum* JZ02	pSSt12	this study
P. rubens			
DSM 1075= Wisconsin 54-1255	wildtype		DSMZ

DSMZ: German Collection of Microorganisms and Cell Cultures GmbH.

Table S3. Oliogonucleotide primers and plasmids used in this study.

Oliogonucleotide primers and plasmids used for genetic manipulation in *A. nidulans*		
		**amplified fragment** (+ overhang to)
**pSSt05**	**expression vector for *A. nidulans* LO8030**	
amp/URA-f	caggggataacgcagg	***amp/URA3***
amp/URA-r	acacaggaaacagctatgac	
SSt31	cgtaatcatggtcatagctgtttcctgtgtctctggaacagtctcgccgt	***wA-PKS-promoter***
SSt32	CATTGTGCAACGCCCTTTGCAGAGCTATCGGgatcaggagaaggagagtcaagtcc	(*amp/URA3, gpdA(p)*)
SSt33	CCGATAGCTCTGCAAAGGGC	***gpdA(p)***
SSt34	catatttcgtcagacacagaataactctcGGCGCCGGTGATGTCTGCTCAAGCGG	(*AfpyrG*)
pyrG-f	gagagttattctgtgtctg	***AfpyrG***
pyrG-r	attctgtctgagaggag	
SSt35	cacgcatcagtgcctcctctcagacagaatcatgacgcgaatcactttactatgac	***wA-PKS-down***
SSt36	ctcacatgttctttcctgcgttatcccctgtgctgtcagtacgcgaagatctc	(*AfpyrG, amp/URA3*)
**pSSt04**	**expression of *xilA* by using pSSt05 as vector**	
SSt39	AACAGCTACCCCGCTTGAGCAGACATCACCGGCatgactgcaccattgccc	***xilA***
SSt04	gctcagtcacttcaccgaca	(*gpdA(p)*)
SSt03	ccagtgtccagcttccgatt	***xilA***
SSt40	catatttcgtcagacacagaataactctcGGCatccagtccatacatcaatgctg	(*AfpyrG*)
Verification of correct gene integration in *A. nidulans* SSt04		
SSt81	gcgagccttccatagttacg	
SSt83	Cagcattctcgaaggcctc	
**pSSt26**	**expression of p*c16g04890* by using pSSt05 as vector**	
SSt137	AGCTACCCCGCTTGAGCAGACATCACCGGCatgactgcaccatggccc	***pc16g04890***
SSt138	ctccaacgttctcttggcaac	(*gpdA(p)*)
SSt139	ggtctcatggcctgcaac	***pc16g04890***
SSt140	tatttcgtcagacacagaataactctcGGCgtctggggaagtggacagaag	(*AfpyrG*)
Verification of correct gene integration in *A. nidulans* SSt26		
SSt81	gcgagccttccatagttacg	
SSt83	cagcattctcgaaggcctc	
**pSSt28**	**expression of *xilC* by using pSSt05 as vector**	
SSt147	CAGCTACCCCGCTTGAGCAGACATCACCGGCatgtcgaggcttgagatagcc	***xilC***
SSt148	tatttcgtcagacacagaataactctcGGCctatcccatgaccaccttgtcc	(*gpdA(p), AfpyrG*)
Verification of correct gene integration in *A. nidulans* SSt28		
SSt81	gcgagccttccatagttacg	
SSt67	tctcgggcatttccaacgtct	

Table S3 continued.

**Oliogonucleotide primers and plasmids used for genetic manipulation of *P. crustosum***		
		**amplified fragment** (+ overhang to)
**pSSt02**	**activation of *xilB* in *P. crustosum* JZ02**	
amp/URA-f	caggggataacgcagg	***Amp/URA3***
amp/URA-r	acacaggaaacagctatgac	
SSt11	cgtaatcatggtcatagctgtttcctgtgtgtcccaatctctagtttgggacag	**1500 bp US of *xilB***
SSt12	acatatttcgtcagacacagaataactctcggtgaaggtgcgaatggttg	(*amp/URA3, AfpyrG*)
pyrG-f	gagagttattctgtgtctg	***AfpyrG***
pyrG-r	attctgtctgagaggag	
SSt07	cacgcatcagtgcctcctctcagacagaatCCGATAGCTCTGCAAAGGGC	***gpdA(p)***
SSt08	GGTGATGTCTGCTCAAGCGG	(*AfpyrG*)
SSt13	AACAGCTACCCCGCTTGAGCAGACATCACCatgcagggccgaaaacg	**first 1500 bp of *xilB***
SSt14	ctcacatgttctttcctgcgttatcccctgtctgggtgggctatagcgaag	(*gpdA(p), amp/URA3*)
Verification of correct gene integration in *P. crustosum* SSt02		
SSt27	caggagaatggcaggctcaa	
SSt28	AATCACCGGCAGTAAGCGAA	
SSt29	cgcgtgtaaaaattgcgtgc	
SSt30	agccgatgcgtcgaaagtat	
**pSSt12**	**activation of *xilA* in *P. crustosum* JZ02**	
amp/URA-f	caggggataacgcagg	***Amp/URA3***
amp/URA-r	acacaggaaacagctatgac	
SSt94	cgtaatcatggtcatagctgtttcctgtgtgaggcattgtttagctgtgtatc	**1500 bp US of *xilA***
SSt95	acatatttcgtcagacacagaataactctcgttgccaatttgtcaaaggtgc	(*Amp/URA3, AfpyrG*)
pyrG-f	gagagttattctgtgtctg	***AfpyrG***
pyrG-r	attctgtctgagaggag	
SSt07	cacgcatcagtgcctcctctcagacagaatCCGATAGCTCTGCAAAGGGC	***gpdA(p)***
SSt08	GGTGATGTCTGCTCAAGCGG	(*AfpyrG*)
SSt96	AACAGCTACCCCGCTTGAGCAGACATCACCatgactgcaccattgccc	**first 1500 bp of *xilA***
SSt97	ctcacatgttctttcctgcgttatcccctgcgttttgtccattcagtcgct	(*gpdA(p), amp/URA3*)
Verification of correct gene integration in *P. crustosum* SSt12		
SSt110	ctgctcgacatgcagagc	
SSt56	tcgggttgtcgtcacatcag	
SSt57	GTTGACAAGGTCGTTGCGTC	
SSt114	gagtgctggtggatgacttg	

Table S4. NMR data of compounds **1**, **2** and **3** in $CDCl_3$.

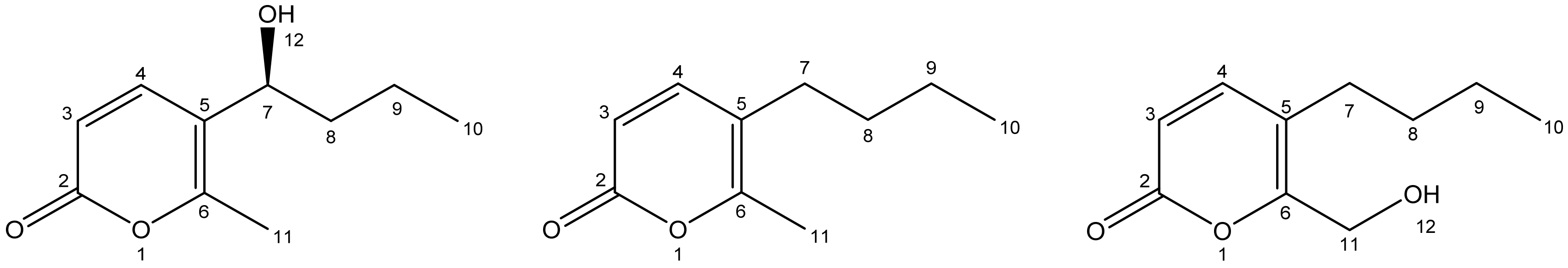

	**1**		**2**		**3**	
	**xylariolide D**		**prexylariolide D**		**xylariolide G**	
**Pos.**	**δ_H (mult., *J* [Hz])**	**δ_C**	**δ_H (mult., *J* [Hz])**	**δ_C**	**δ_H (mult., *J* [Hz])**	**δ_C**
**2**		162.4		163.0		161.8
**3**	6.21 (d, 9.6)	114.0	6.14 (d, 9.5)	113.3	6.27 (d, 9.5)	115.7
**4**	7.48 (d, 9.6)	143.3	7.16 (d, 9.5)	147.1	7.22 (d, 9.5)	146.9
**5**		118.3		115.5		116.1
**6**		158.3		158.3		157.5
**7**	4.64 (t, 6.9)	68.3	2.28 (t, 7.7)	29.2	2.36 (t, 7.7)	28.6
**8**	1.74, 1.53 (m)	39.4	1.45 (m)	32.0	1.47 (m)	32.5
**9**	1.39, 1.28 (m)	19.1	1.34 (m)	22.2	1.34 (m)	22.2
**10**	0.95 (t, 7.3)	13.9	0.93 (t, 7.3)	13.9	0.93 (t, 7.3)	13.9
**11**	2.26 (s)	17.1	2.22 (s)	17.2	4.45 (s)	59.0
**12**	not observed		-		not observed	

The NMR data of **1** and **2** correspond well to those reported previously [7,8].

Table S5. Enrichments and coupling constants in **1** and **2** after feeding with ^{13}C labeled precursors.

Feeding with		sodium [2-^{13}C] acetate	sodium [1,2-^{13}C] acetate			sodium [2-^{13}C] acetate	sodium [1,2-^{13}C] acetate	
		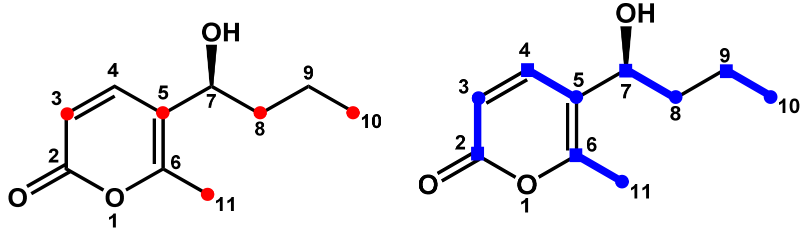				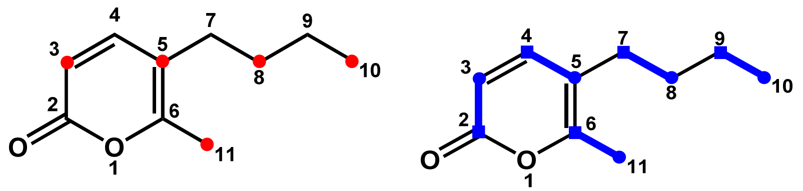		
		xylarioloide D (**1**)				prexylarioloide D (**2**)		
**Pos.**	**δC**	**enrichment**	**enrichment**	**J [Hz]**	**δC**	**enrichment**	**enrichment**	**J [Hz]**
**2**	162.4	1.2	3.4	72.6	163.0	1.0	3.9	72.4
**3**	114.0	6.7	3.5	72.6	113.3	9.6	3.3	72.4
**4**	143.3	1.0	3.4	52.8	147.1	1.0	3.0	51.8
**5**	118.3	5.1	2.7	52.8	115.5	5.5	3.0	51.8
**6**	158.3	1.1	3.5	52.4	158.3	1.0	3.7	53.1
**7**	68.3	0.8	3.3	37.9	29.2	0.8	3.3	33.6
**8**	39.4	7.0	3.1	37.9	32.0	8.6	3.4	33.6
**9**	19.1	0.9	4.3	34.7	22.2	1.0	3.3	34.7
**10**	13.9	7.5	3.9	34.7	13.9	9.5	4.3	34.7
**11**	17.1	5.6	3.9	52.4	17.2	6.6	4.0	53.1

Supplementary Figures

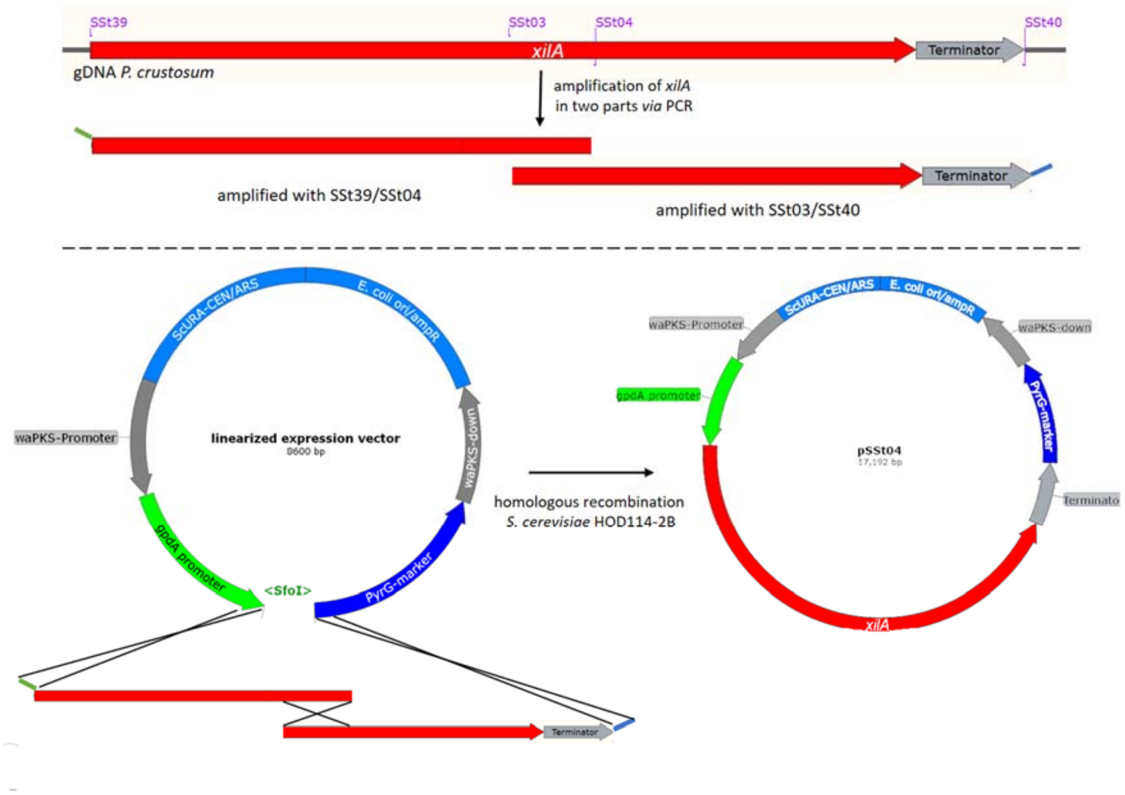

Figure S1. Schematic representation of plasmid creation.
Creation of plasmid pSSt04. *XilA* was amplified *via* PCR from gDNA of *P. crustosum* PRB-2 in two fragments. Subsequent transformation of *S. cerevisiae* HOD114-2B with the linearized empty vector and the two DNA fragments of *xilA* was used for homologous recombination.

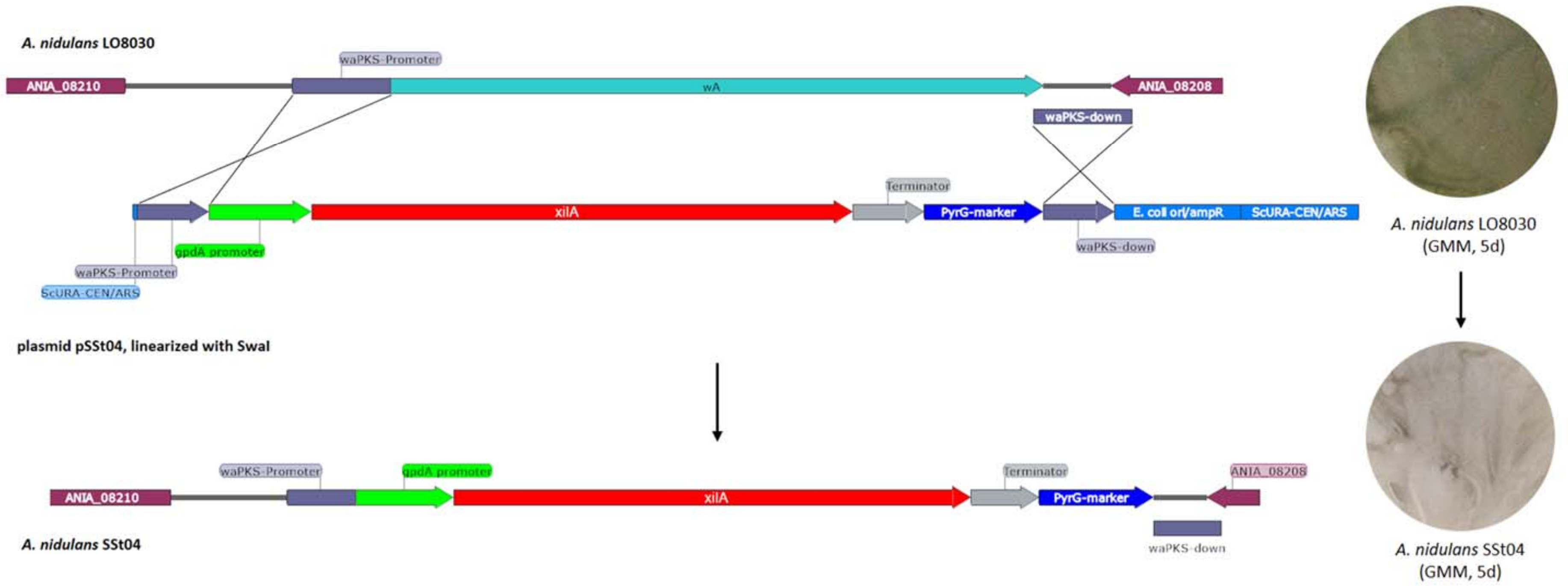

Figure S2. Schematic representation of gene integration into the *wA* PKS locus of *A. nidulans* LO8030.
In *A. nidulans* LO8030, the *wa* PKS gene is responsible for the formation of YWA1, a yellow pigment found in mature sexual spores [9]. During homologous recombination, the *wA* gene is replaced by the gene to be expressed. Successful integration at the *wA* locus leads to transformants with a white phenotype, whereas transformants with etopic integration remain green.

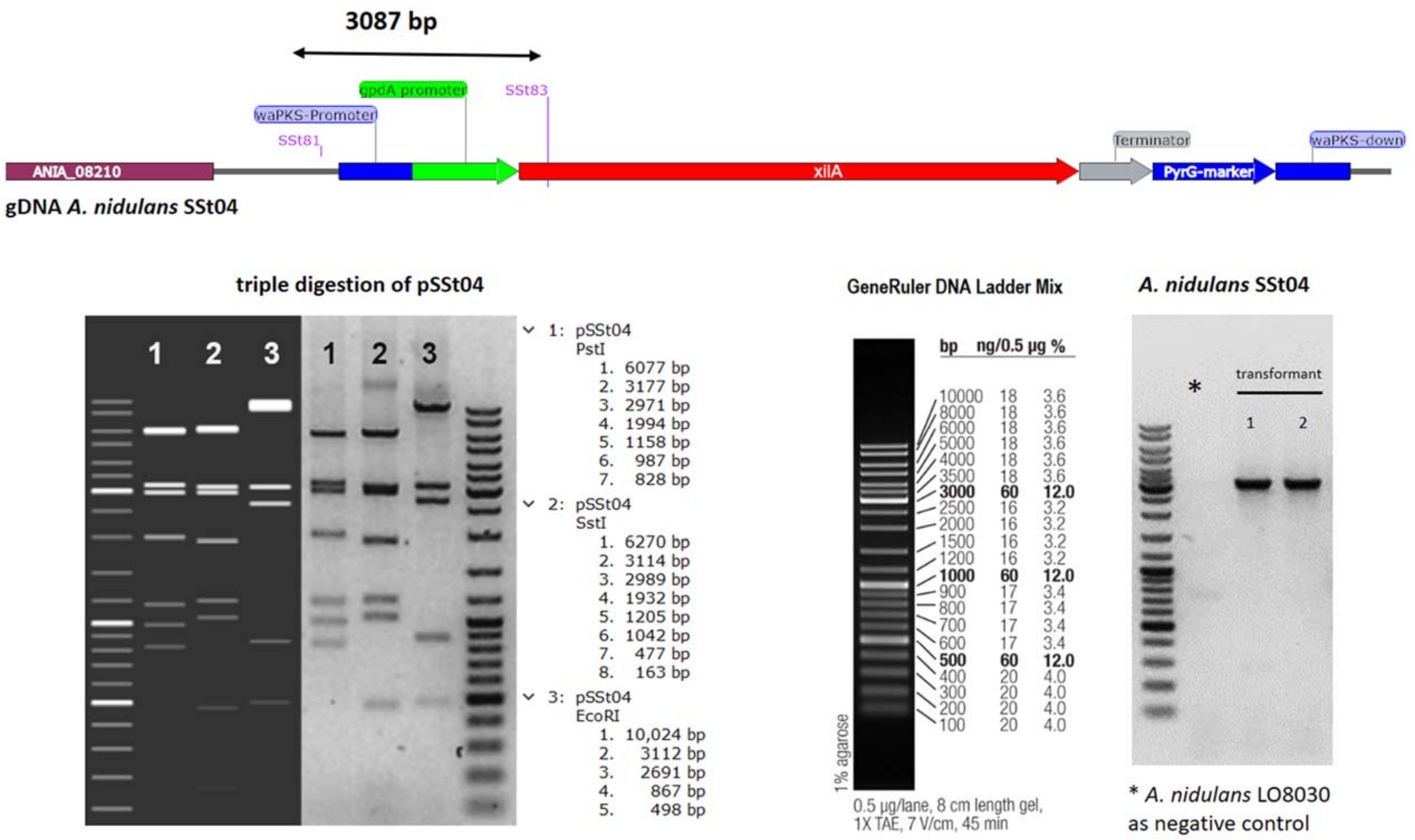

Figure S3. Verification of plasmid pSSt04 and correct integration in *A. nidulans* SSt04 transformants.
GeneRuler DNA Ladder Mix from Thermo Fisher was used as a size standard for DNA fragments. Verification of transformants *via* PCR was performed with primers binding outside of the integration construct in genomic DNA and inside of integrated genes. Verification of pSSt04 was carried out by triple digestion with restriction enzymes *Pst*I, *Sst*I, and *EcoR*I.

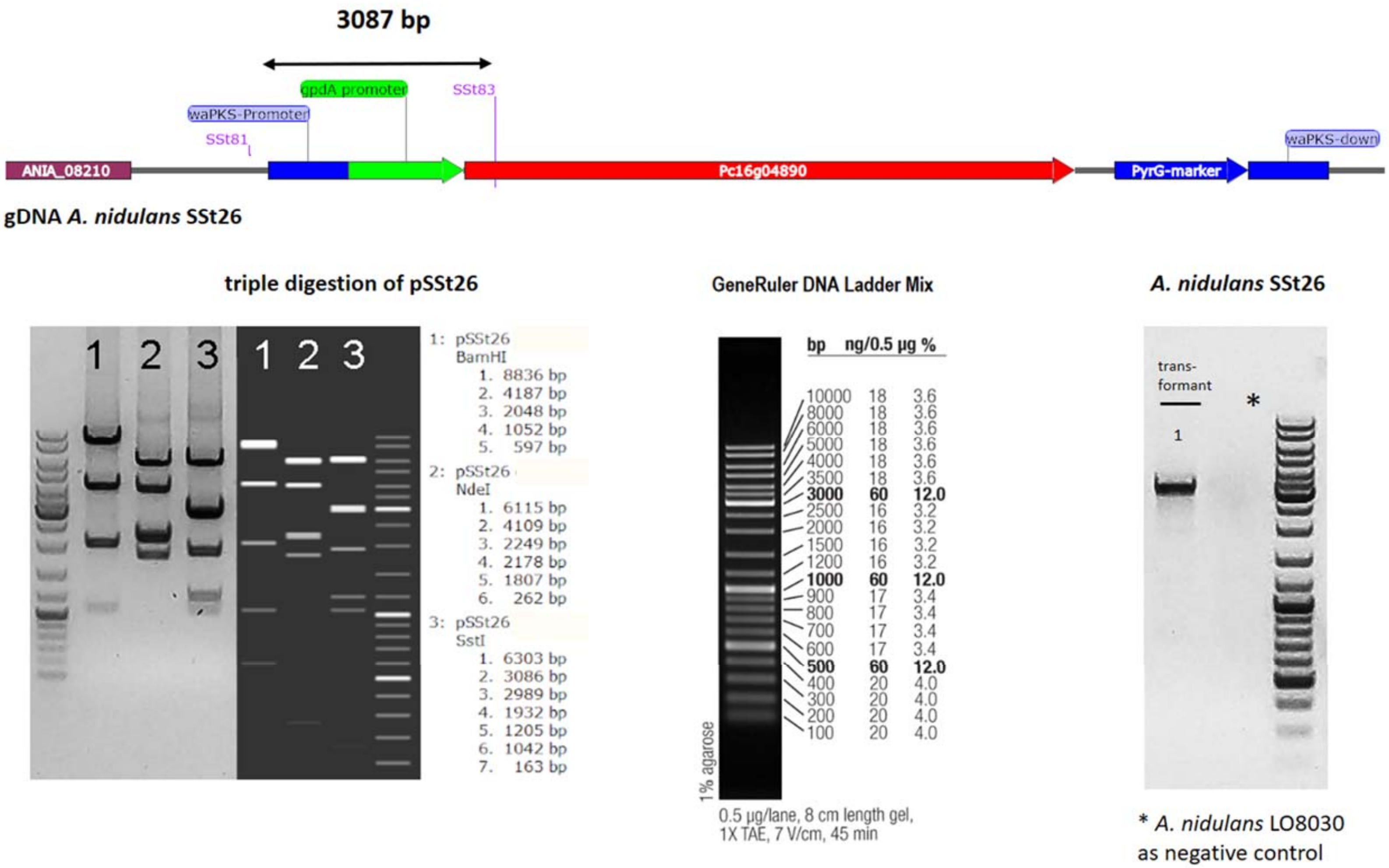

Figure S4. Verification of plasmid pSSt26 and correct integration in *A. nidulans* SSt26 transformant.
Verification of pSSt26 was carried out by triple digestion with restriction enzymes *BamH*I, *Nde*I, and *Sst*I. Due to the similar structure of PKS *pc16g04890* from *P. rubens* Wisconsin 54-1255, the same primers used for *A. nidulans* SSt04 could also be used to verify transformant for *A. nidulans* SSt26.

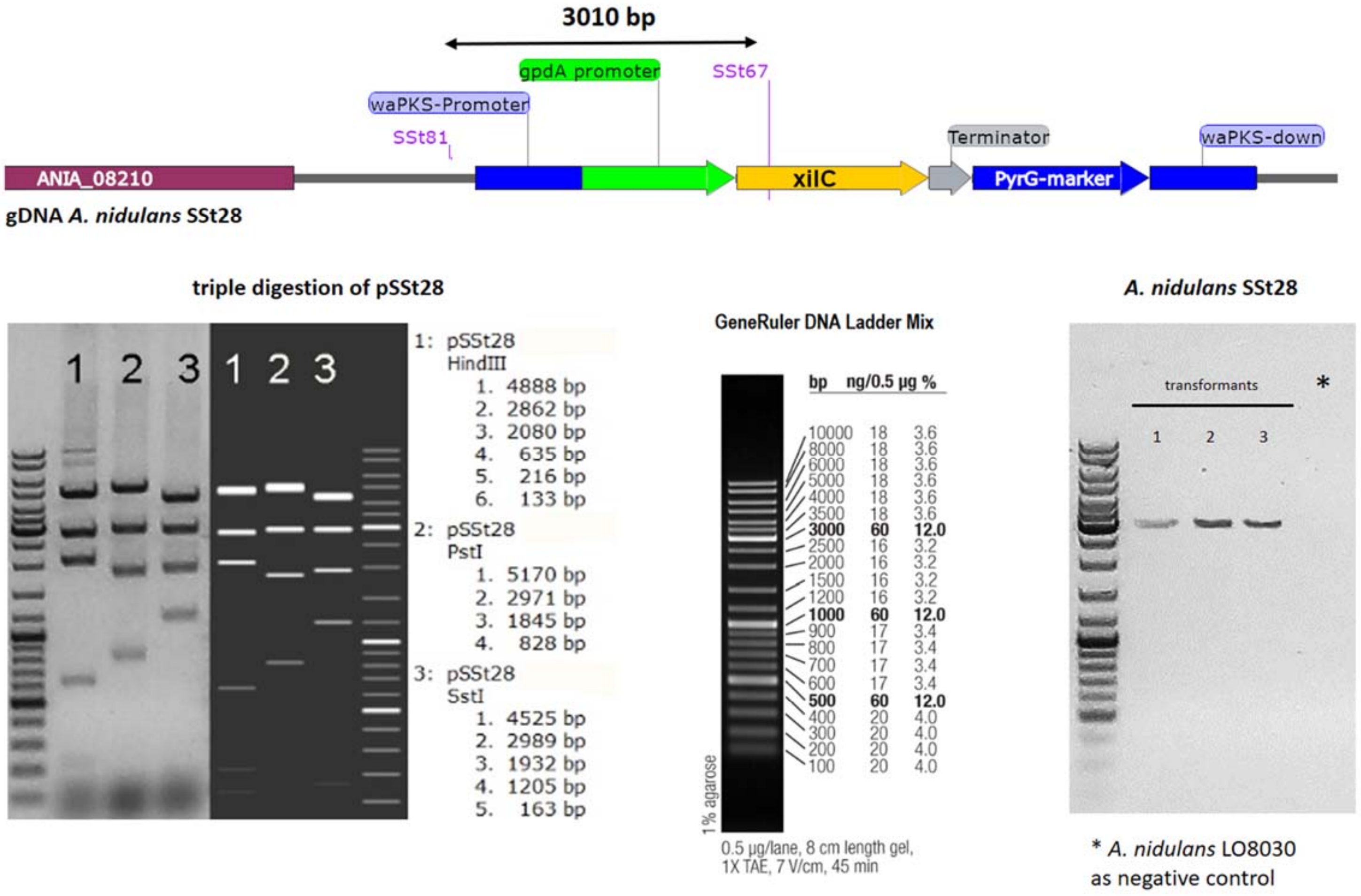

Figure S5. Verification of plasmid pSSt28 and correct integration in *A. nidulans* SSt28 transformants.
Verification of pSSt28 was carried out by triple digestion with restriction enzymes *HInd*III, *Pst*I, and *Sst*I.

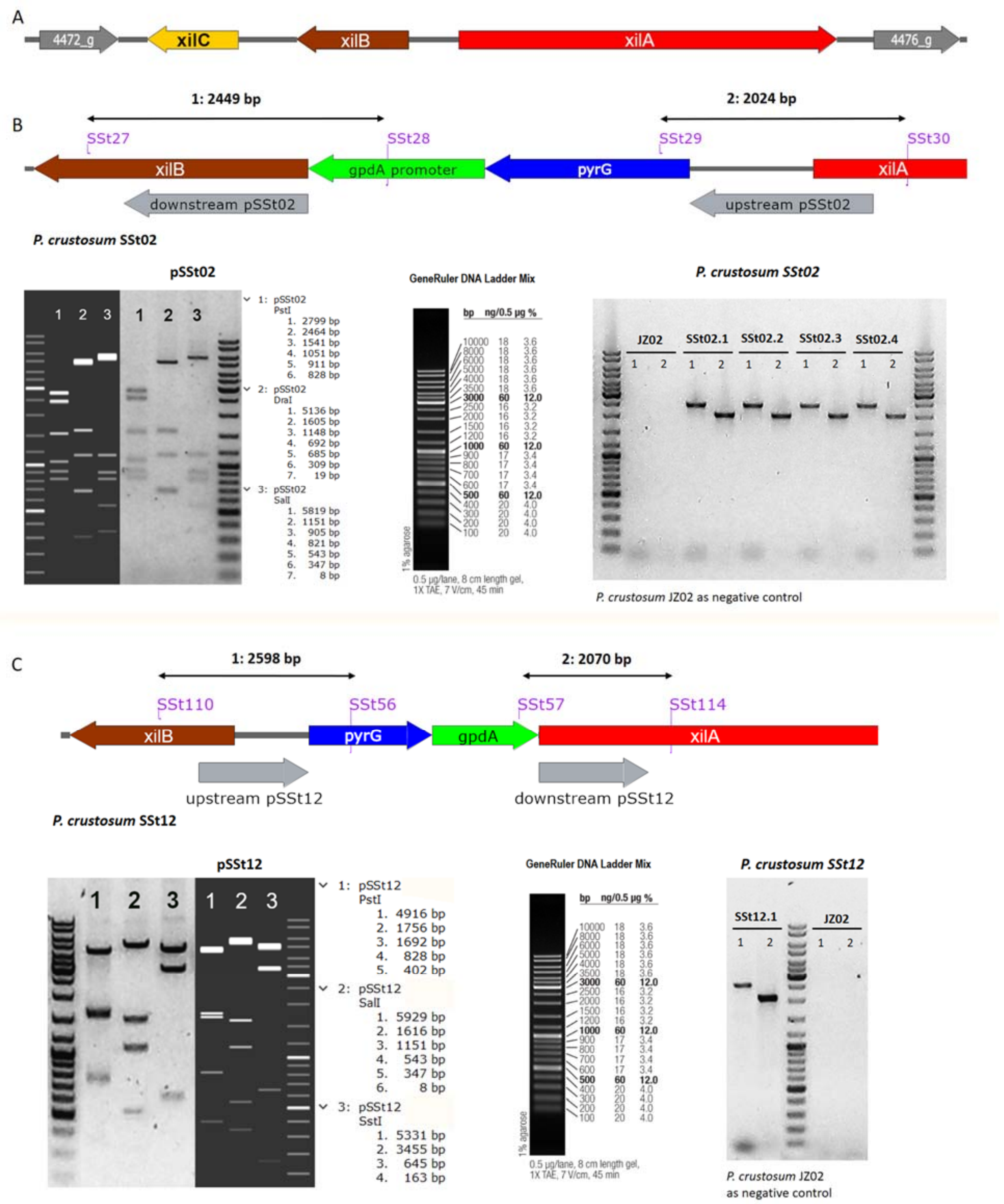

Figure S6. Verification of plasmids and correct gene integration in *P. crustosum* transformants.

- **A.** Genomic region of the *xil* cluster in *P. crustosum* PRB-2.
- **B.** Verification of plasmid pSSt02 and transformants for *P. crustosum* SSt02
- **C.** Verification of plasmid pSSt12 and transformant for *P. crustosum* SSt12.

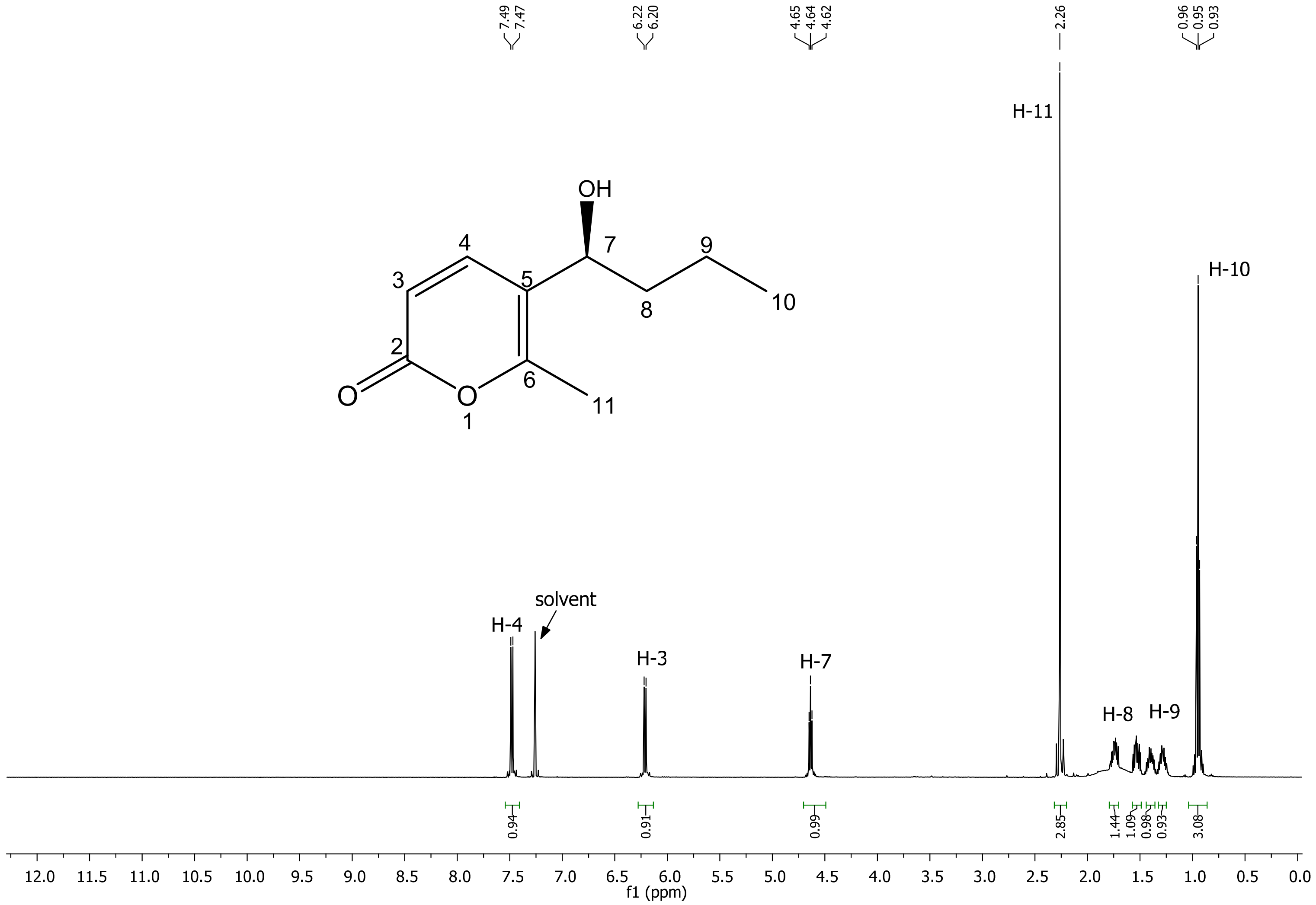

Figure S7. ^{1}H-NMR of compound **1** in $CDCl_3$ (500 MHz).

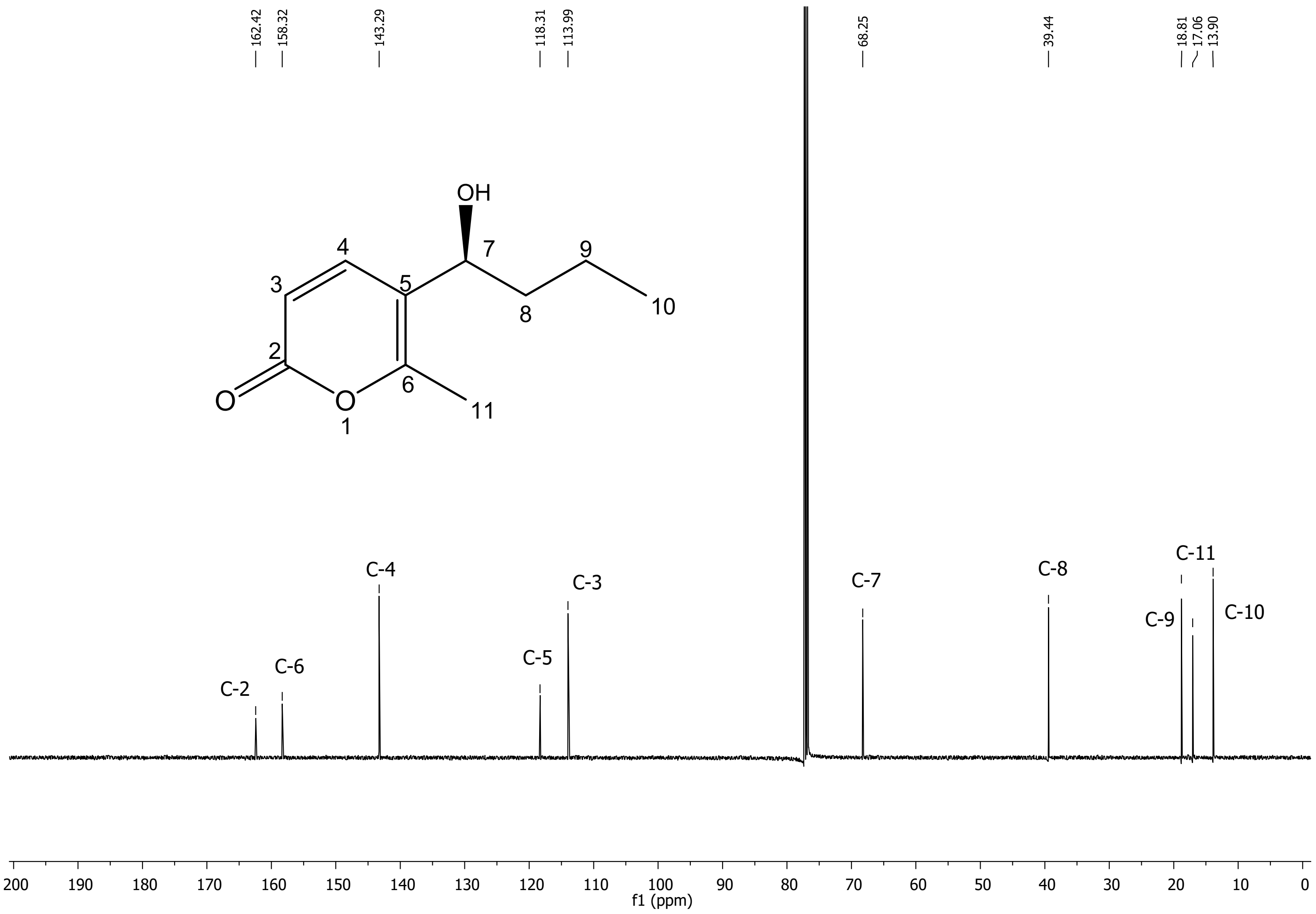

Figure S8. ^{13}C-NMR of compound **1** in $CDCl_3$ (125 MHz).

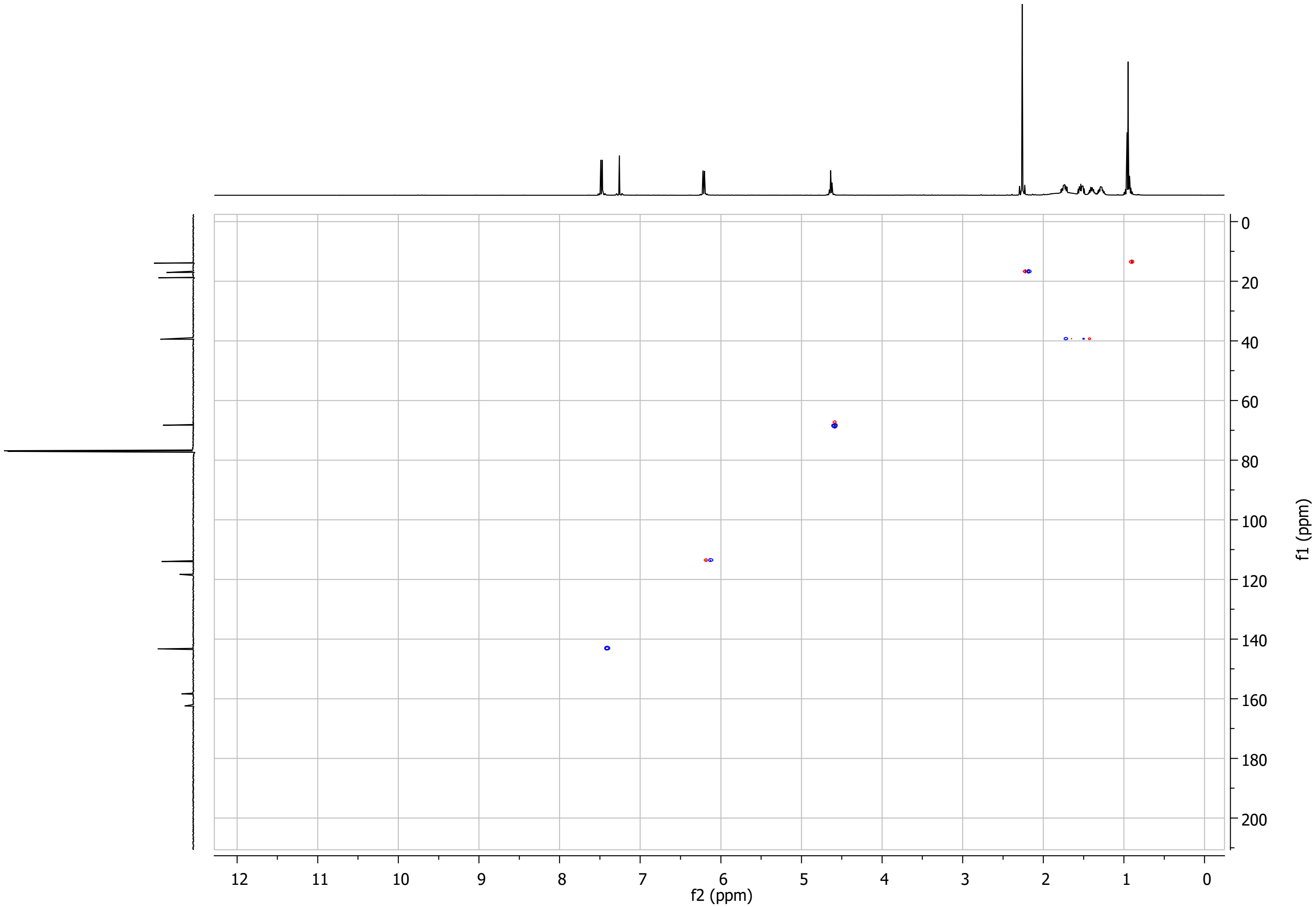

Figure S9. HSQC of compound **1** in $CDCl_3$.

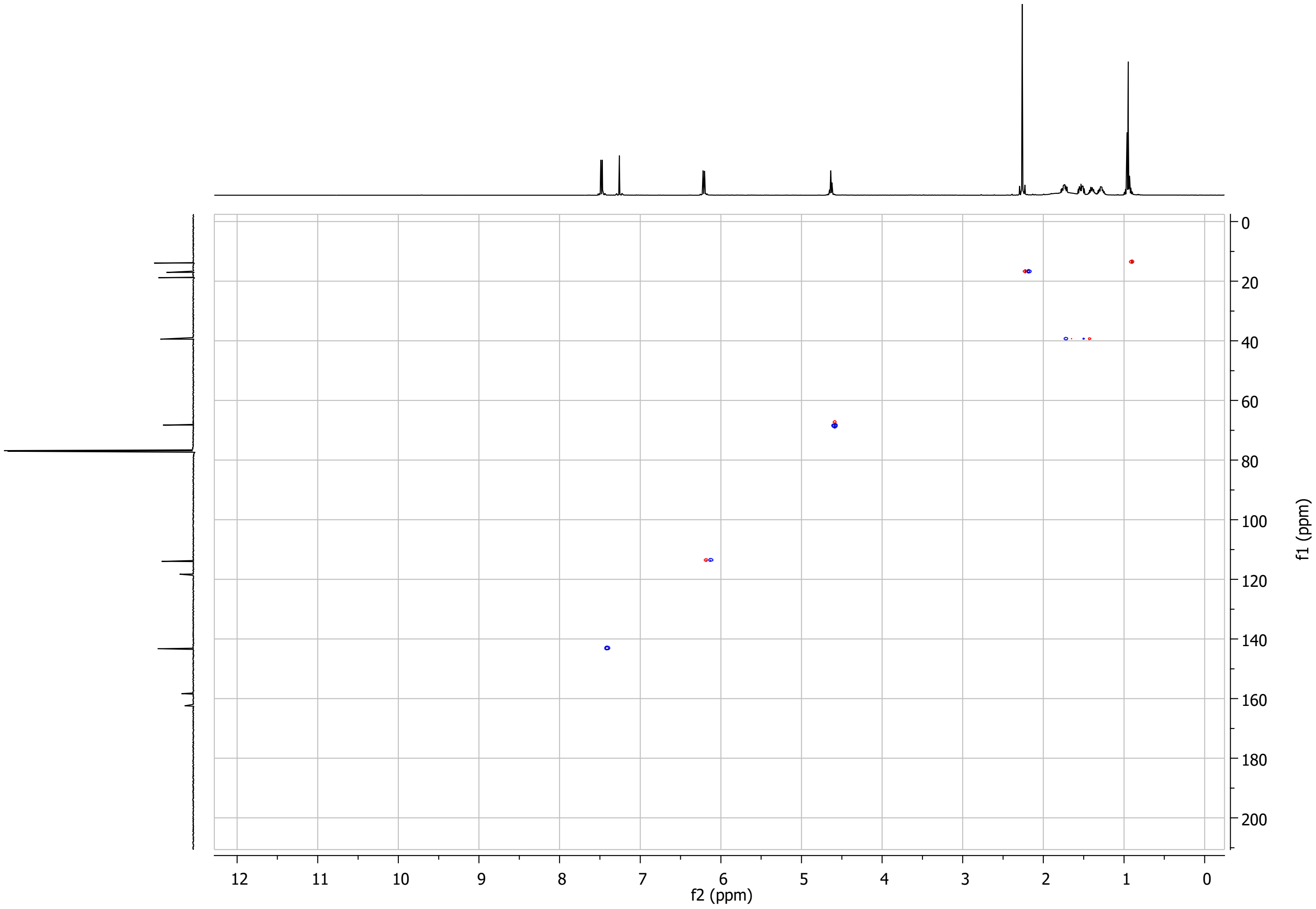

Figure S10. HMBC of compound **1** in $CDCl_3$.

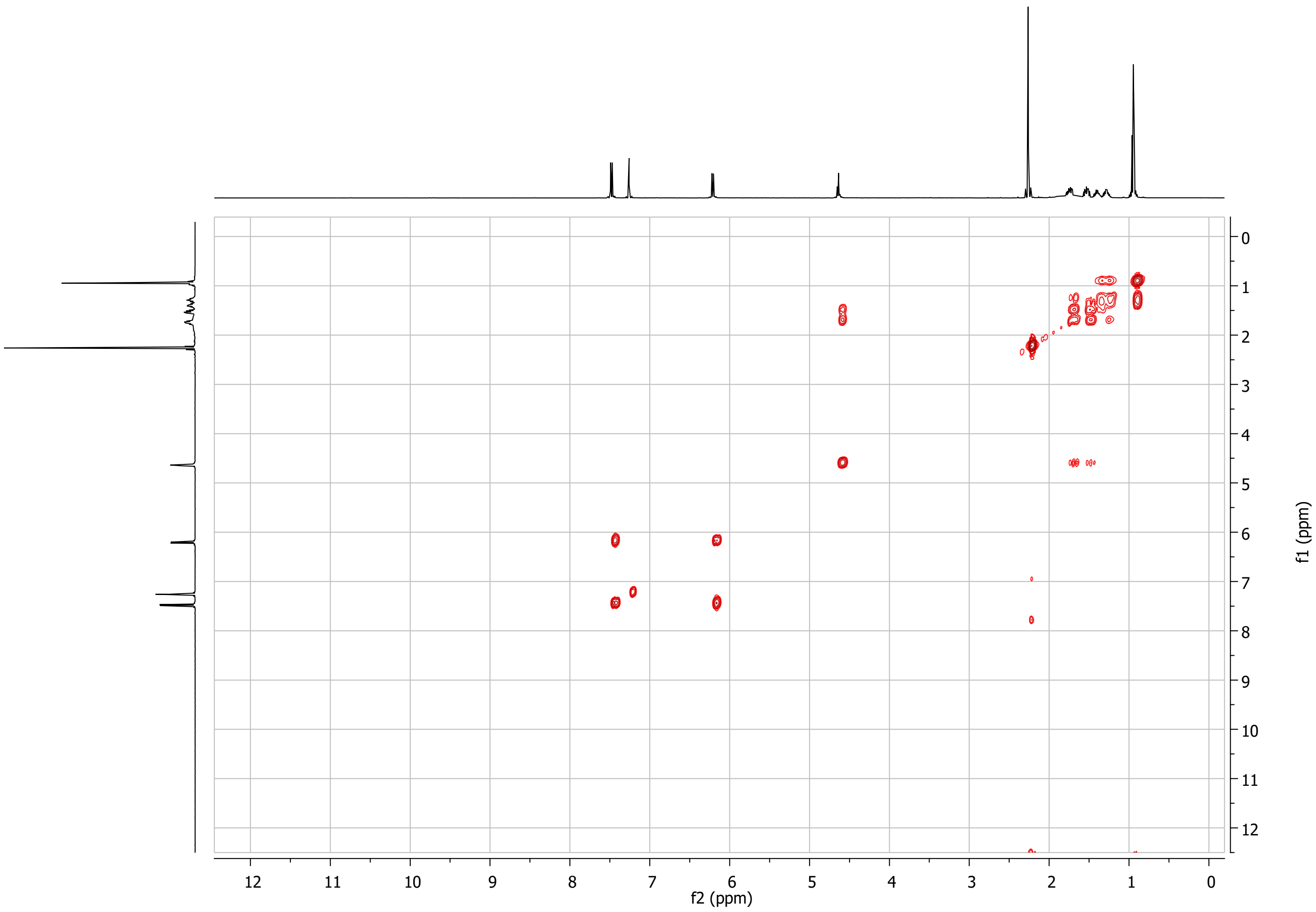

Figure S11. H,H-COSY of compound **1** in $CDCl_3$.

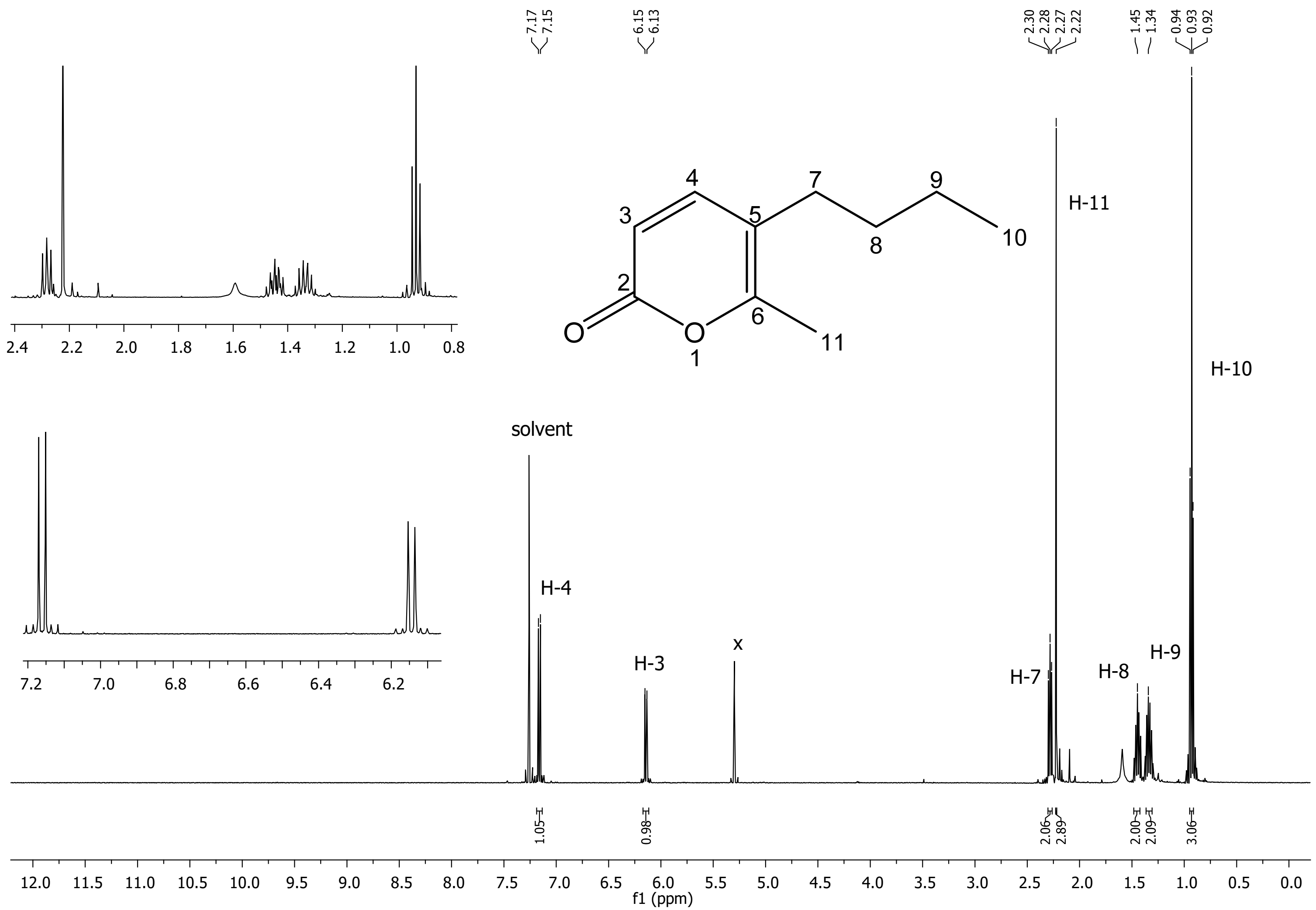

Figure S12. ^{1}H-NMR of compound **2** in $CDCl_3$ (500 MHz).

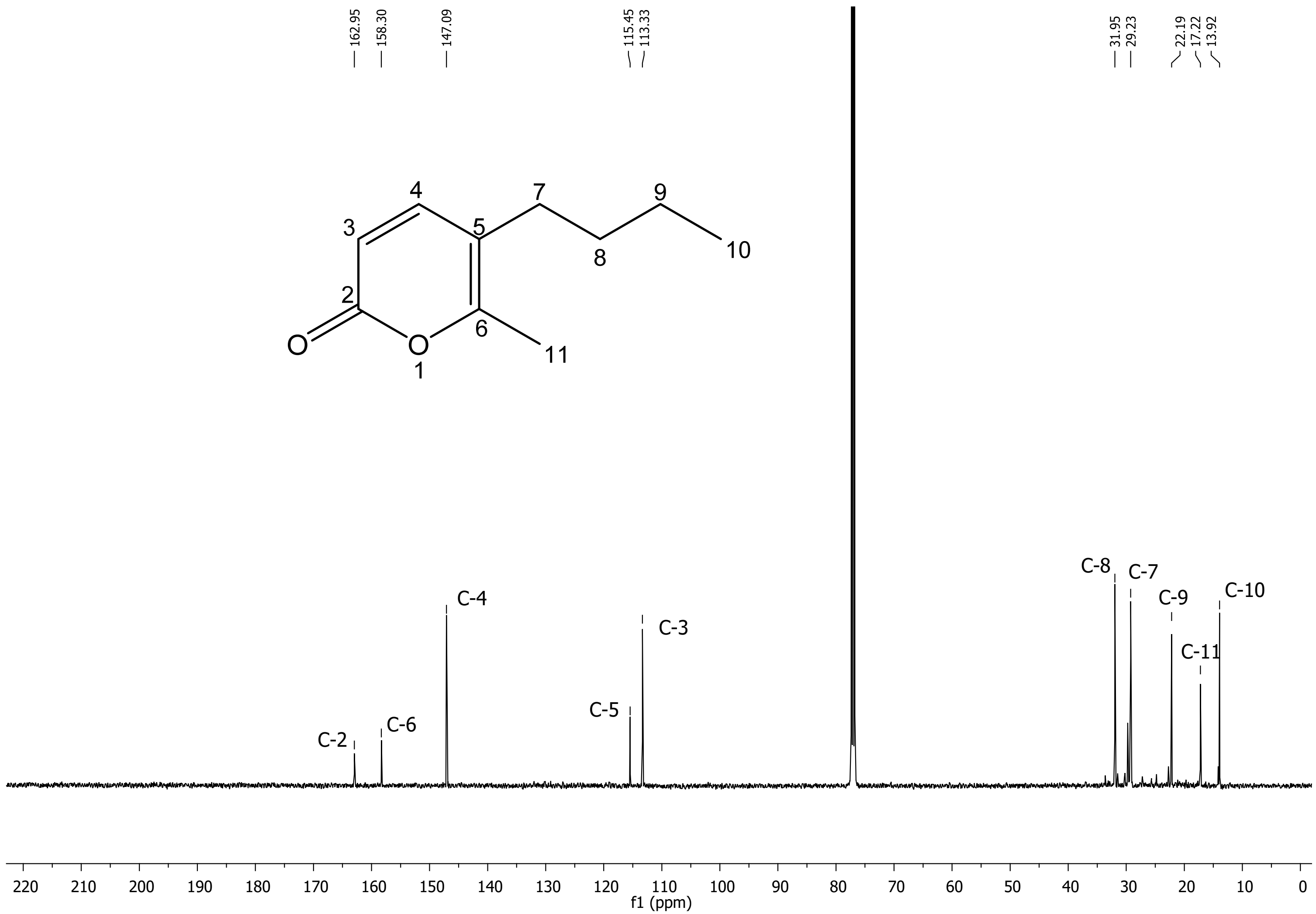

Figure S13. ^{13}C-NMR of compound **2** in $CDCl_3$ (125 MHz).

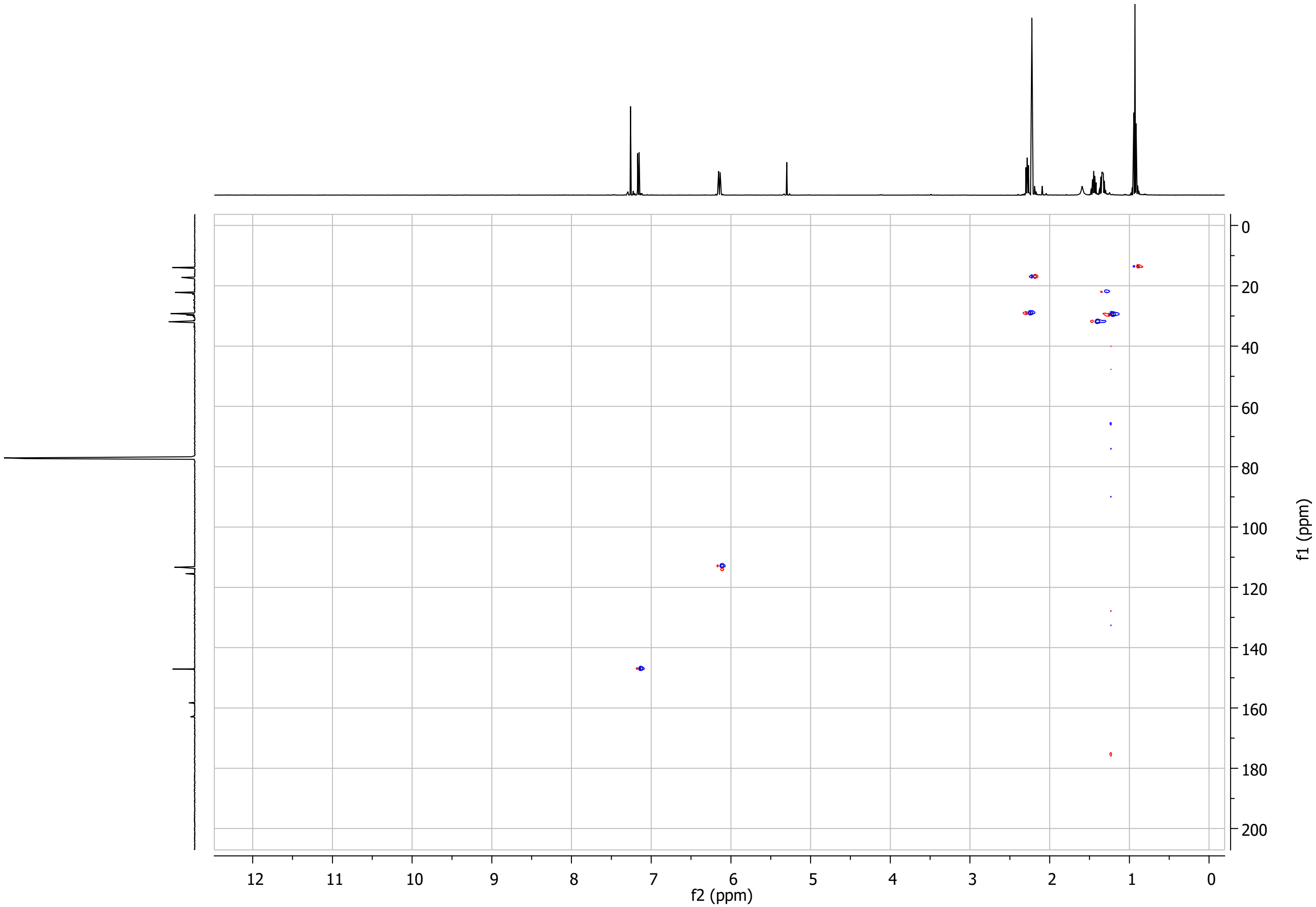

Figure S14. HSQC of compound **2** in $CDCl_3$.

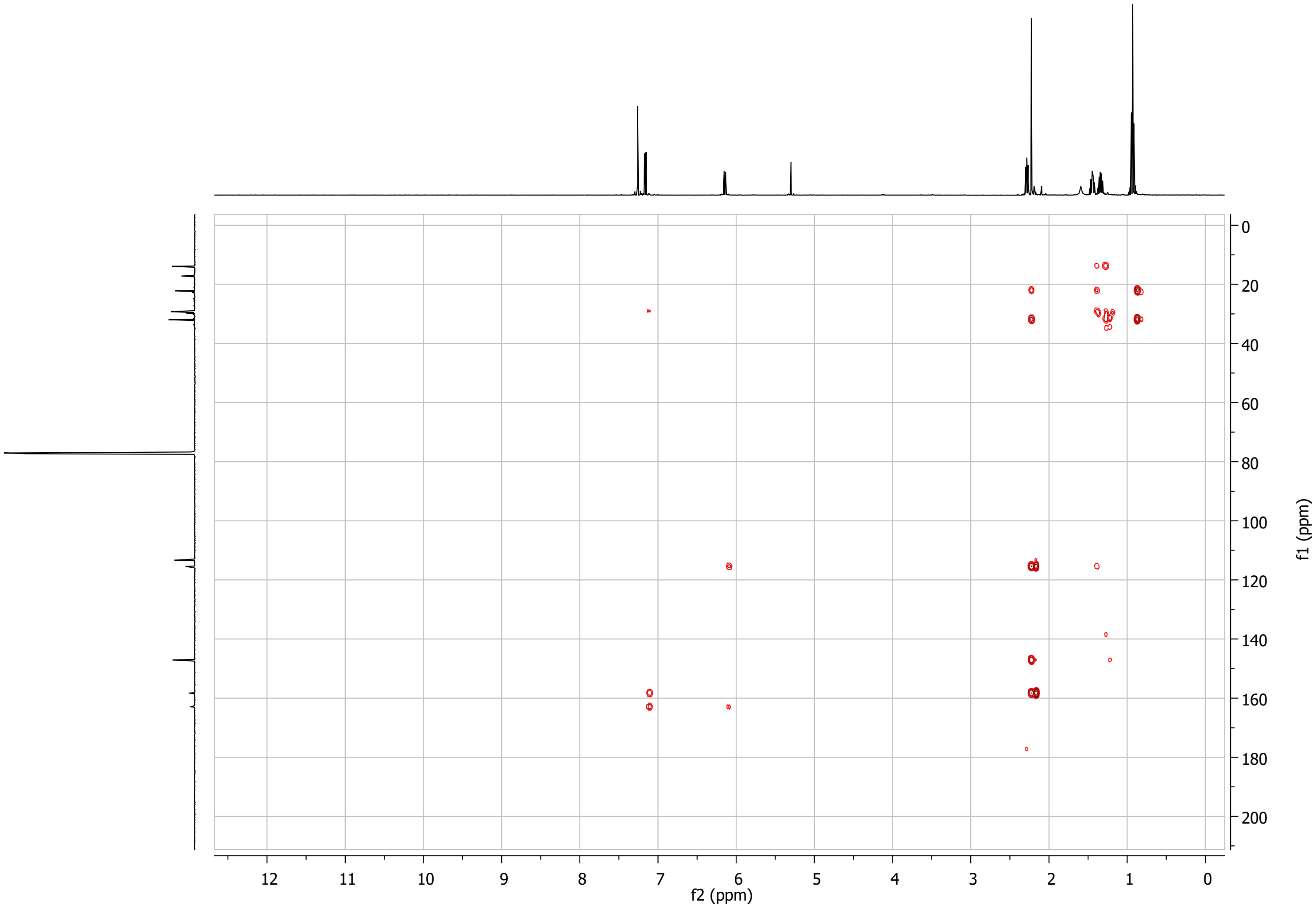

Figure S15. HMBC of compound **2** in $CDCl_3$.

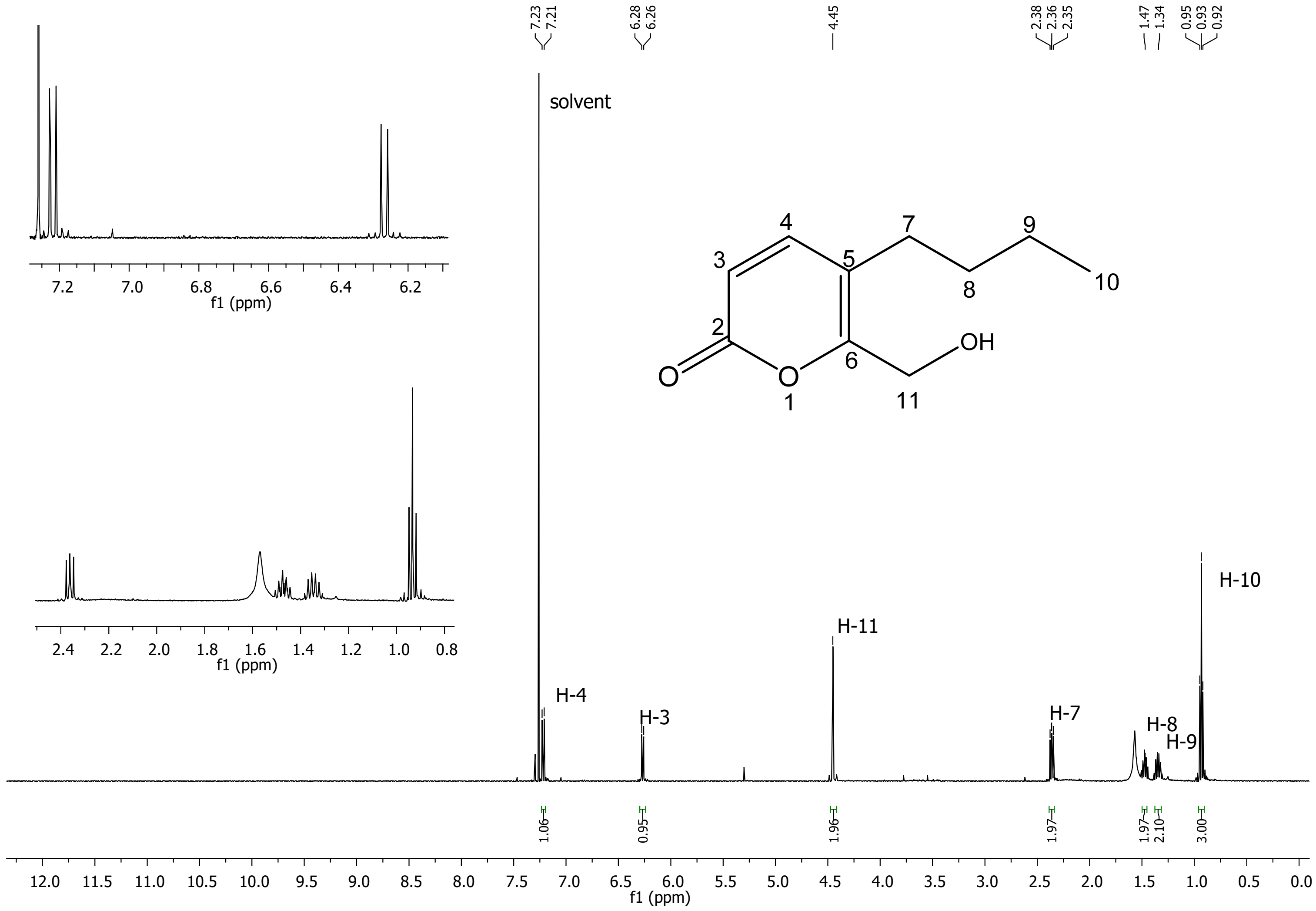

Figure S16. ^{1}H-NMR of compound **3** in $CDCl_3$ (500 MHz).

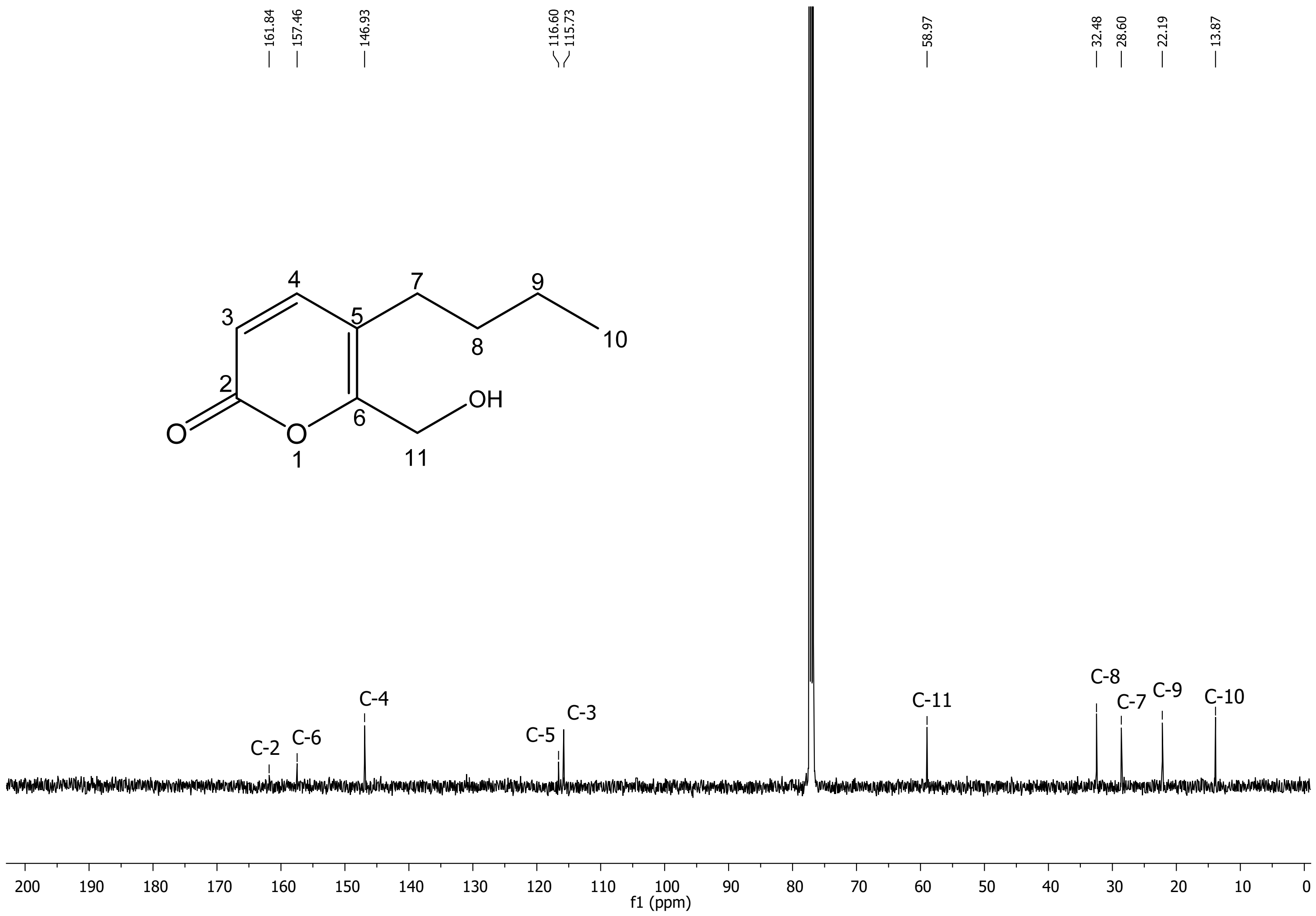

Figure S17. ^{13}C-NMR of compound **3** in $CDCl_3$ (125 MHz).

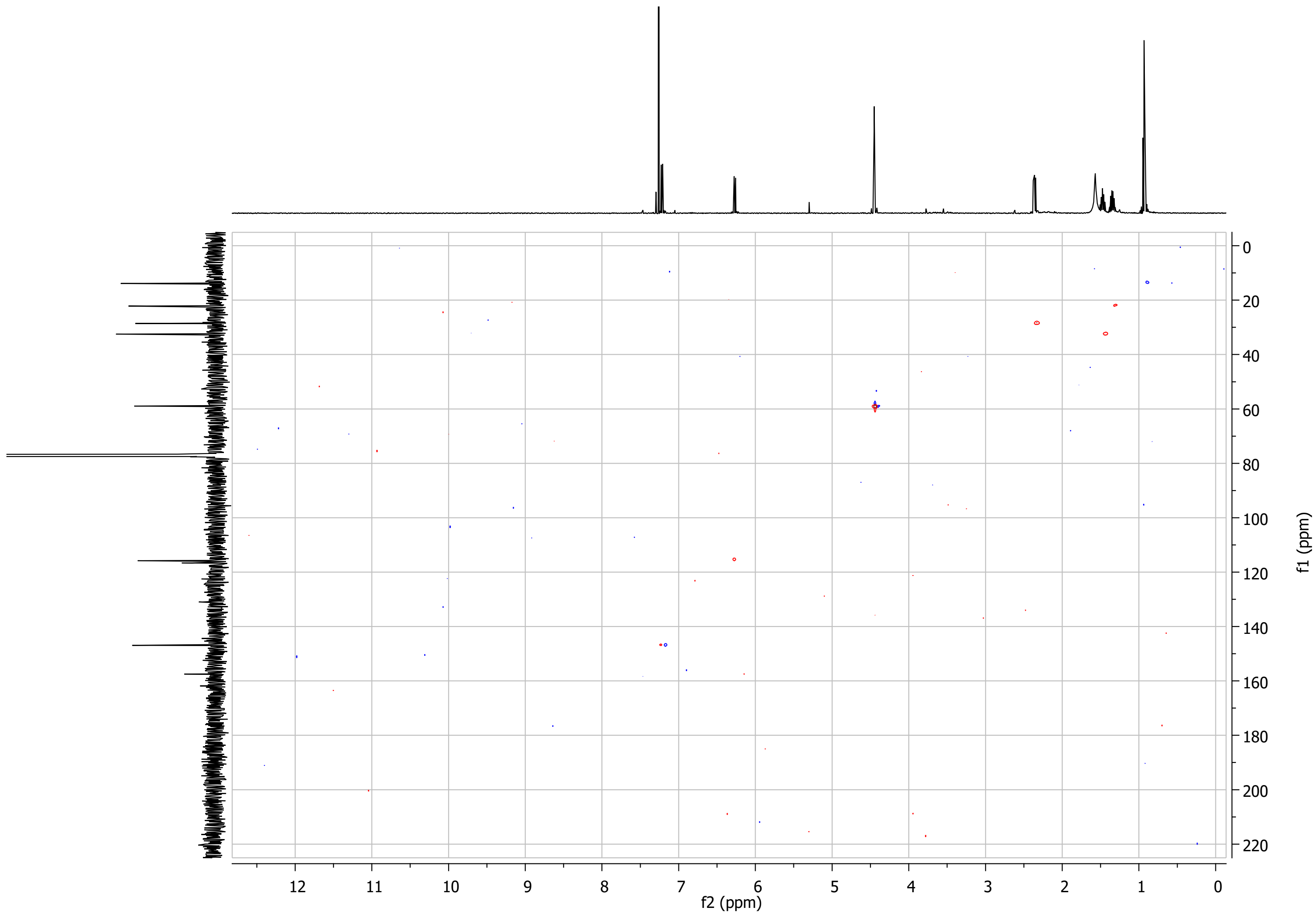

Figure S18. HSQC of compound **3** in $CDCl_3$.

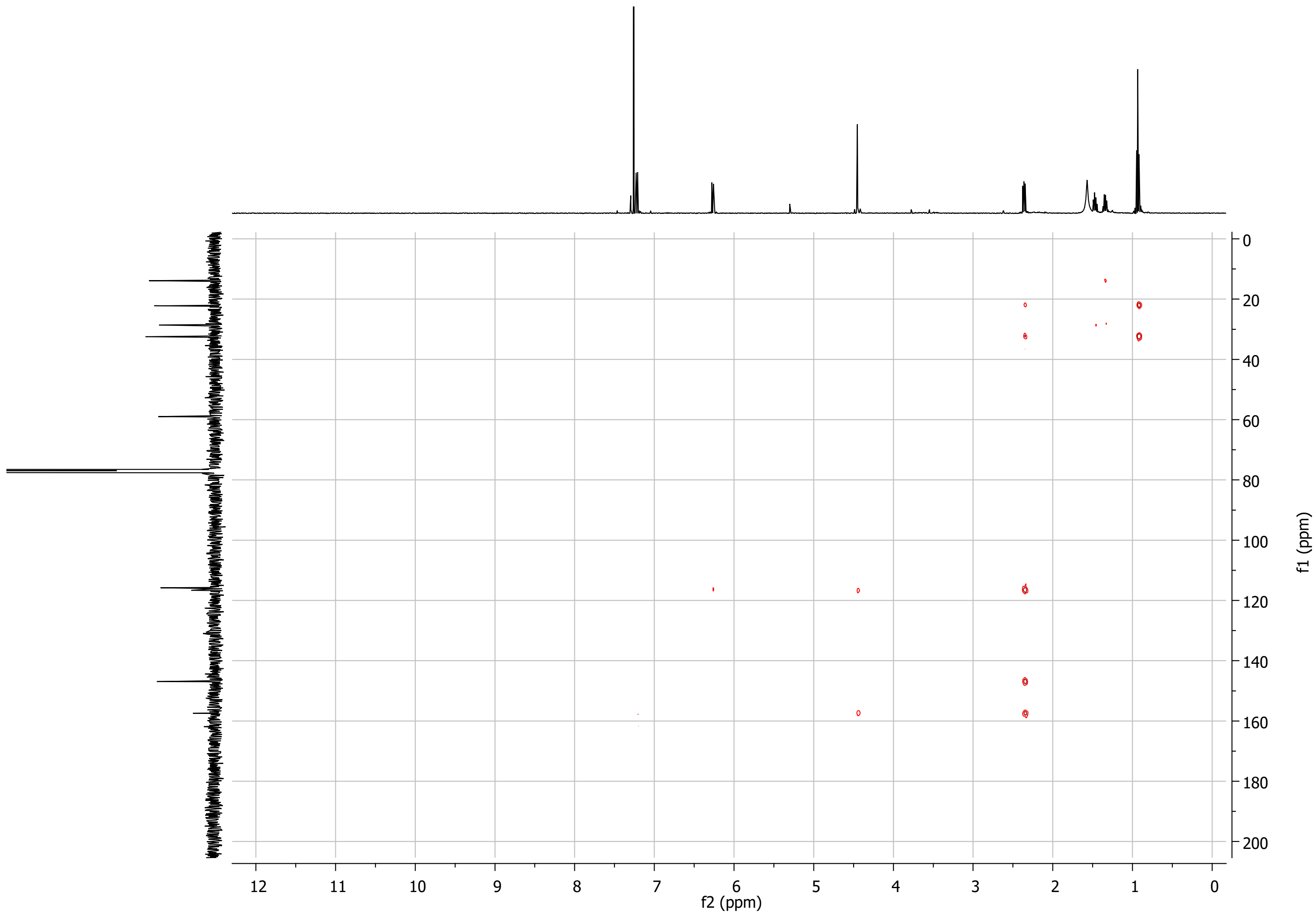

Figure S19. HMBC of compound **3** in $CDCl_3$.

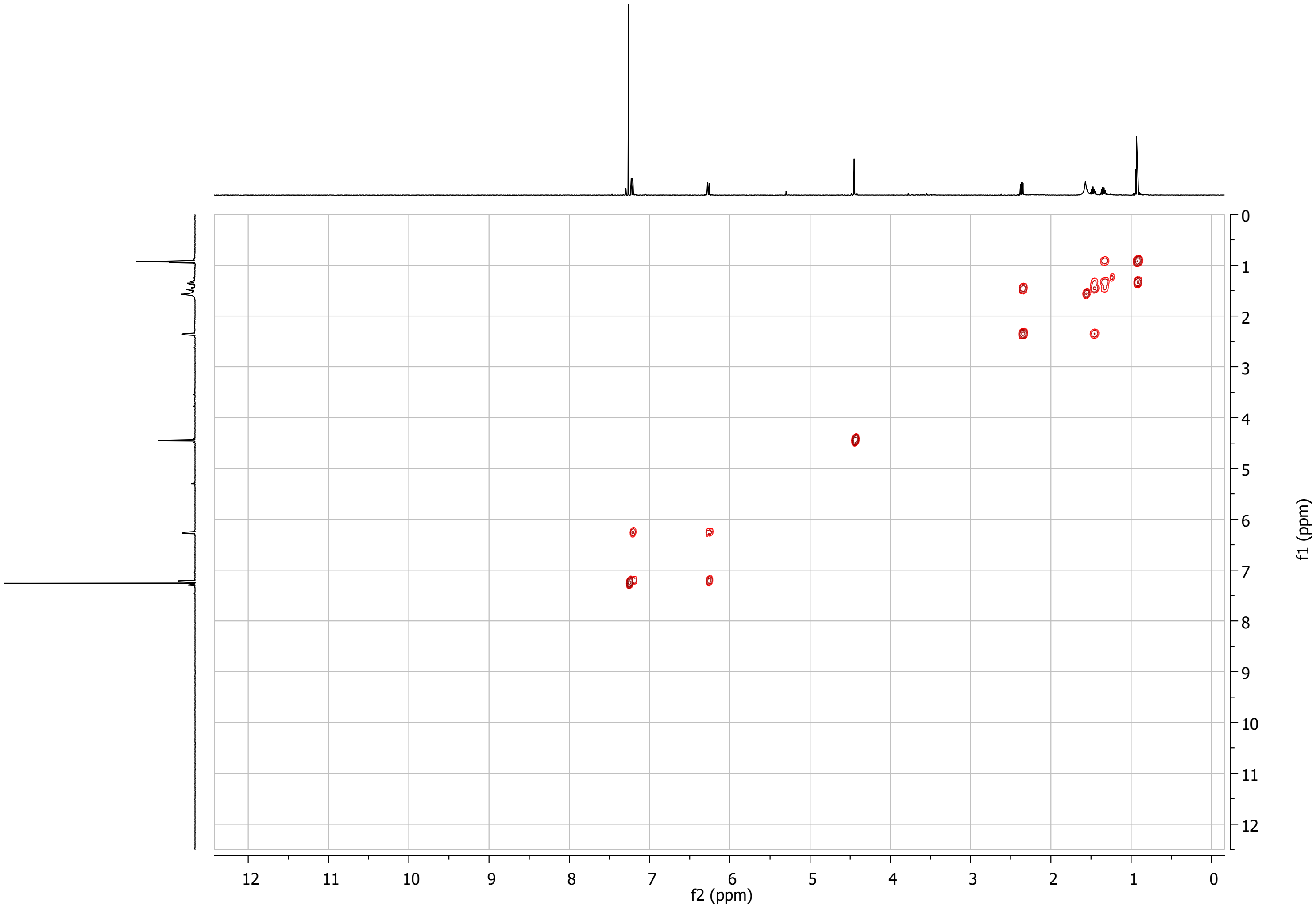

Figure S20. H,H-COSY of compound **3** in $CDCl_3$.

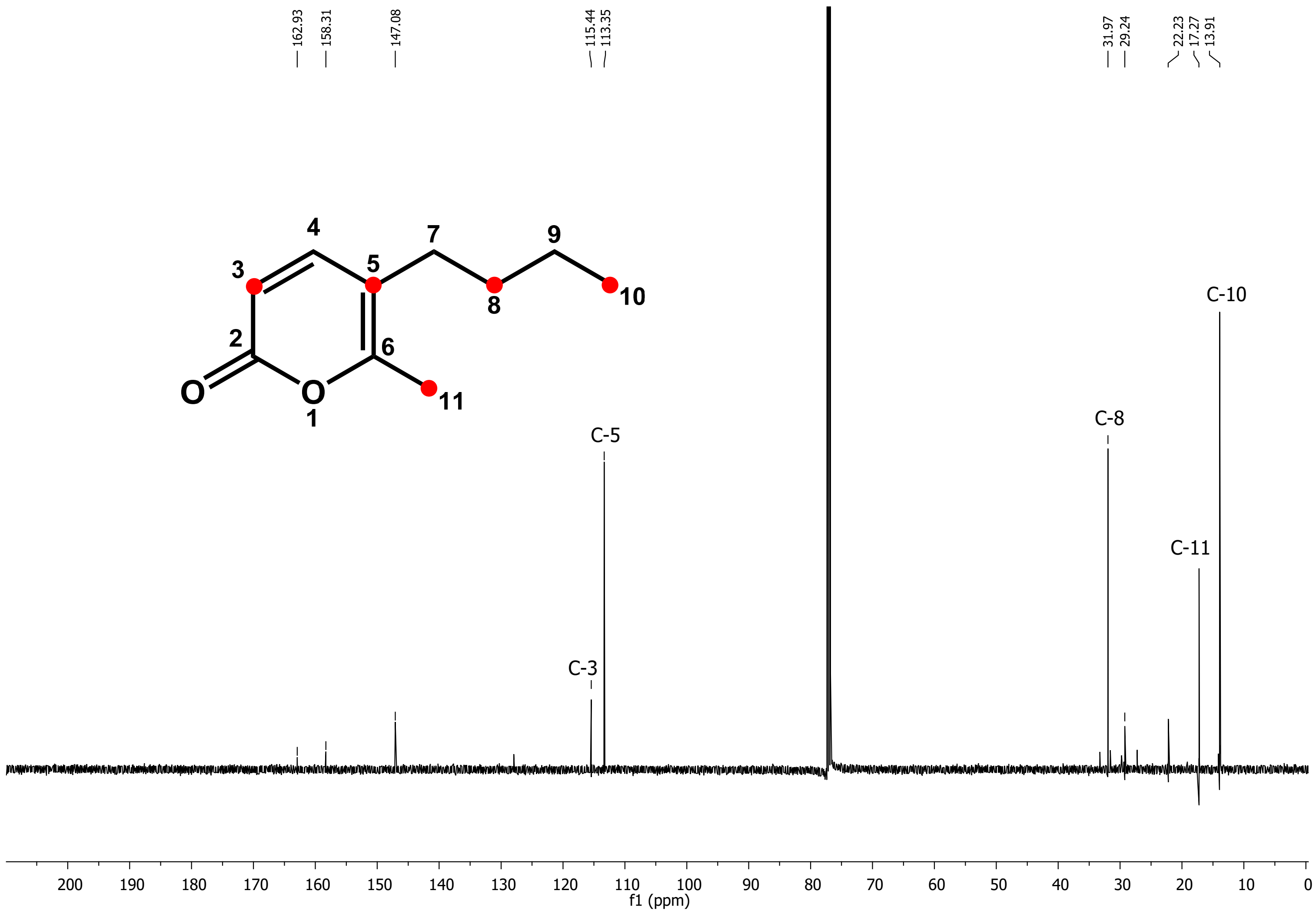

Figure S21. ^{13}C-NMR of compound **2** in $CDCl_3$, enriched with [2-^{13}C] acetate (125 MHz).

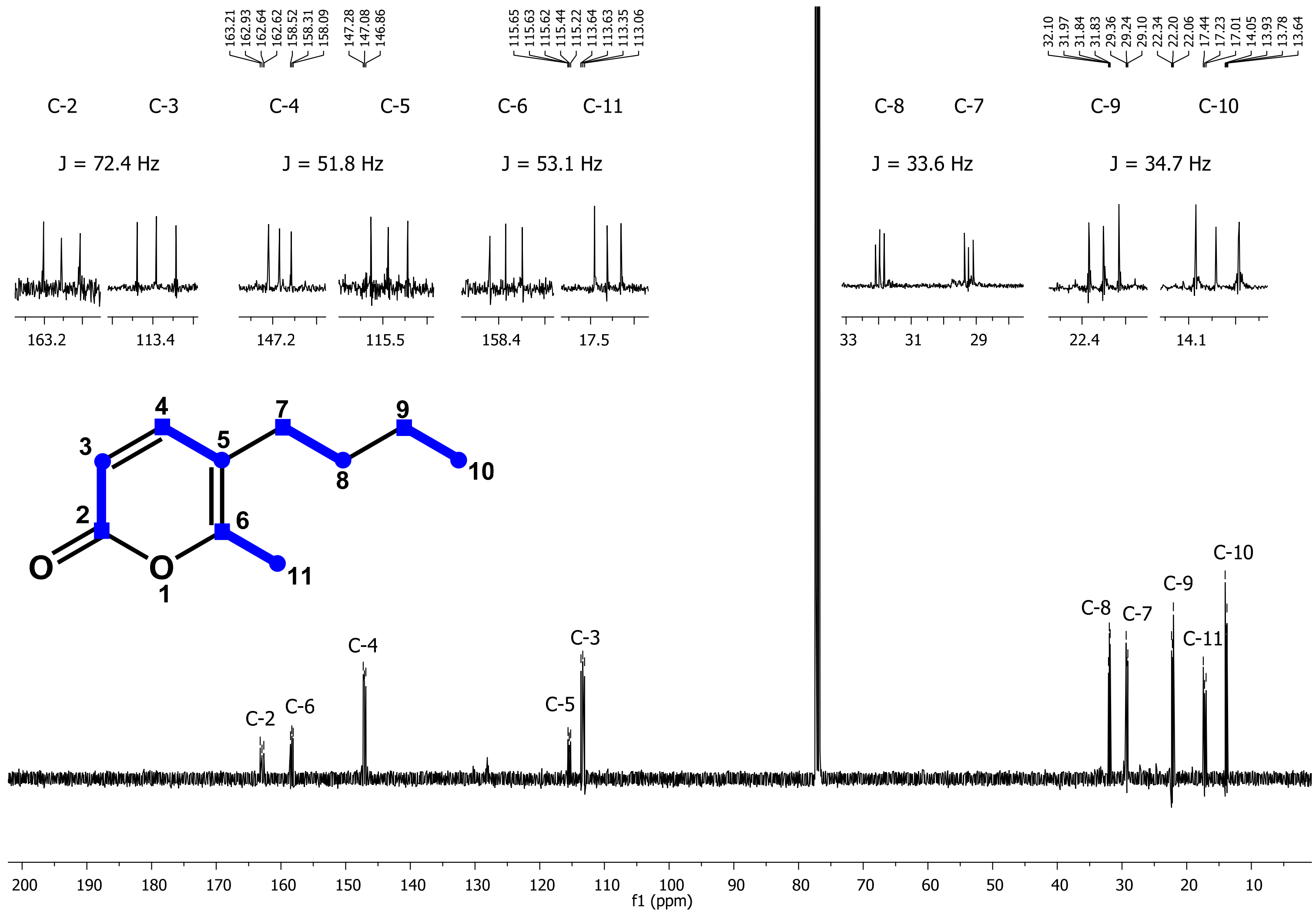

Figure S22. ^{13}C-NMR of compound **2** in $CDCl_3$, enriched with [1,2-^{13}C] acetate (125 MHz).

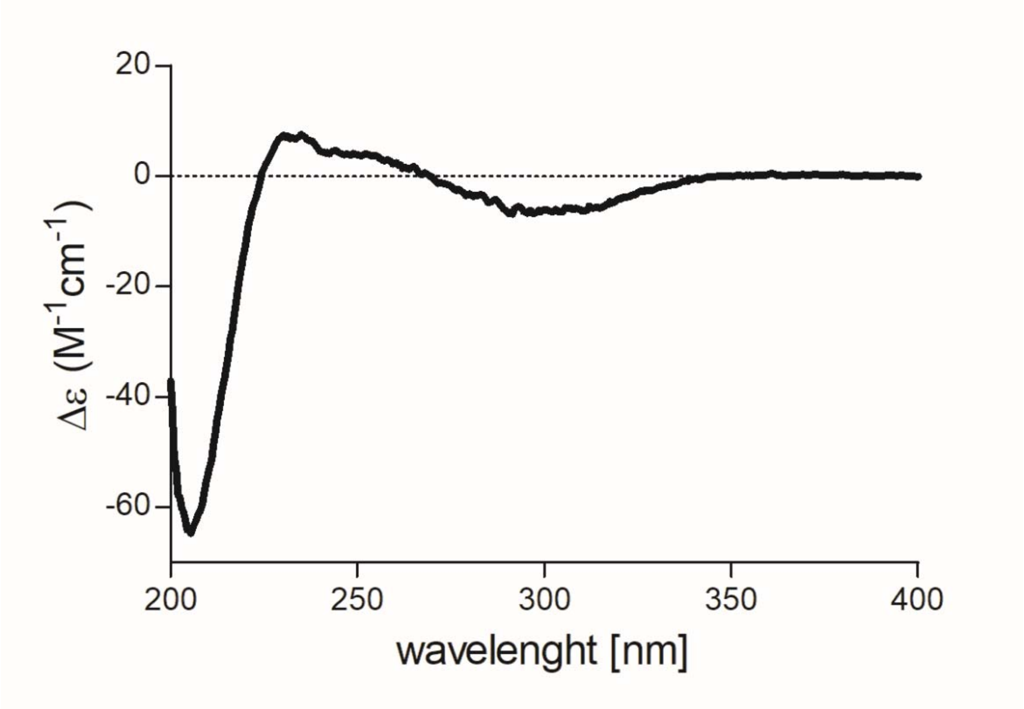

Figure S23. ECD-spectrum of compound **1** (0.7 mg/mL) in MeOH.

References

1. Blin, K.; Shaw, S.; Kloosterman, A. M.; Charlop-Powers, Z.; Van Wezel, G. P.; Medema, M. H.; Weber, T. antiSMASH 6.0: improving cluster detection and comparison capabilities. *Nucleic Acids Res.* **2021**, *49,* W29.
2. Keller, N. P. Fungal secondary metabolism: regulation, function and drug discovery. *Nat. Rev. Microbiol.* **2019**, *17,* 167.
3. Mojardín, L.; Vega, M.; Moreno, F.; Schmitz, H. P.; Heinisch, J. J.; Rodicio, R. Lack of the NAD^+-dependent glycerol 3-phosphate dehydrogenase impairs the function of transcription factors Sip4 and Cat8 required for ethanol utilization in *Kluyveromyces lactis*. *Fungal. Genet. Biol.* **2018**, *111,* 16.
4. Chiang, Y. M.; Ahuja, M.; Oakley, C. E.; Entwistle, R.; Asokan, A.; Zutz, C.; Wang, C. C.; Oakley, B. R. Development of genetic dereplication strains in *Aspergillus nidulans* results in the discovery of aspercryptin. *Angew. Chem. Int. Ed. Engl.* **2016**, *55,* 1662.
5. Wu, G.; Ma, H.; Zhu, T.; Li, J.; Gu, Q.; Li, D. Penilactones A and B, two novel polyketides from Antarctic deep-sea derived fungus *Penicillium crustosum* PRB-2. *Tetrahedron* **2012**, *68,* 9745.
6. Zhou, J. and Li, S.-M. Conversion of viridicatic acid to crustosic acid by cytochrome P450 enzyme-catalysed hydroxylation and spontaneous cyclisation. *App. Microbol. Biotechnol.* **2021**, *105,* 9181.
7. Hu, Z.-Y.; Li, Y.-Y.; Lu, C.-H.; Lin, T.; Hu, P.; Shen, Y.-M. Seven novel linear polyketides from *Xylaria* sp. NCY2. *Helv. Chim. Acta* **2010**, *93,* 925.
8. Schlingmann, G.; Milne, L.; Carter, G. T. New α-pyrones produced by fungal culture LL-11G219 function as androgen receptor ligands. *Tetrahedron* **1998**, *54,* 13013.
9. Watanabe, A.; Fujii, I.; Sankawa, U.; Mayorga, M. E.; Timberlake, W. E.; Ebizuka, Y. Re-identification of *Aspergillus nidulans* wA gene to code for a polyketide synthase of naphthopyrone. *Tetrahedron Lett.* **1999**, *40,* 91.
